# Dysregulation of the CD147 complex confers defective placental development: A pathogenesis of early‐onset preeclampsia

**DOI:** 10.1002/ctm2.826

**Published:** 2022-06-02

**Authors:** Cheuk‐Lun Lee, Zhilong Chen, Qingqing Zhang, Yue Guo, Vivian W.Y. Ng, Baozhen Zhang, Kunfeng Bai, Degong Ruan, Anita S.Y. Kan, Ka‐Wang Cheung, Annisa S.L. Mak, William S.B. Yeung, Rui Su, Qing Yang, Min Chen, Mei‐Rong Du, Zhang Jian, Xiujun Fan, Philip C.N. Chiu

**Affiliations:** ^1^ Shenzhen Key Laboratory of Fertility Regulation The University of Hong Kong‐Shenzhen Hospital Shenzhen China; ^2^ Department of Obstetrics and Gynaecology LKS Faculty of Medicine The University of Hong Kong Hong Kong China; ^3^ Center for Energy Metabolism and Reproduction Institute of Biomedicine and Biotechnology Shenzhen Institutes of Advanced Technology, Chinese Academy Sciences Shenzhen China; ^4^ College of Veterinary Medicine Hunan Agricultural University Changsha China; ^5^ University of Chinese Academy of Sciences Beijing China; ^6^ Li Ka Shing Faculty of Medicine School of Biomedical Sciences Stem Cell & Regenerative Medicine Consortium The University of Hong Kong Hong Kong China; ^7^ Department of Obstetrics and Gynecology Queen Elizabeth Hospital Hong Kong China; ^8^ Department of Fetal Medicine and Prenatal Diagnosis The Third Affiliated Hospital of Guangzhou Medical University Guangzhou China; ^9^ NHC Key Lab of Reproduction Regulation (Shanghai Institute of Planned Parenthood Research) Hospital of Obstetrics and Gynecology Fudan University Shanghai Medical College Shanghai China

Dear Editor,

Trophoblastic CD147, also known as basigin (BSG), regulates the differentiation and spiral artery remodelling functions of extravillous trophoblasts (EVCTs), and its deficiency contributes to the pathophysiology of preeclampsia (PE). The biological activities of CD147 are mediated by its interaction with integrin β1 and Wnt/β‐catenin signalling. The reduced serum and villous CD147 levels in PE are worth further investigation in a clinical trial as an early biomarker of PE.

PE is a multifactorial gestational complication affecting 4.6% of pregnancies worldwide.^1^ It is the top cause of prenatal morbidity/mortality and is associated with a high incidence of maternal and perinatal complications, causing a heavy burden to the healthcare system.^1^ The aetiology of PE is associated with defective trophoblast differentiation and functions causing abnormal placental development, insufficient placental perfusion, and maternal–foetal exchange defects.^1^, ^2^ Our limited knowledge of the pathogenesis of the disease has hindered the development of a reliable approach for the prediction and treatment of PE.

CD147 is a component of several of the most abundant protein complexes in human and mouse placentas^3^ (Figure S1). It has been linked to the physiology and pathology of various reproductive processes.^4^ Systematic CD147 knockout leads to perinatal lethality before day 12.5 of gestation in mice.^5^ The observations could be due to deficiency of CD147 in the foetus and/or the placenta. To circumvent the limitation of the knockout model, we employed our nanoparticle model^6^ to specifically knockdown CD147 expression in mouse trophoblasts (Figure S2). Our results showed that trophoblastic CD147 knockdown caused PE‐like symptoms in mice, including placental haemorrhage (Figure 1A), reduction of alive litter size (Figure 1B), reduction of body, placental and foetal weight (Figure 1C‐E), and elevation of blood pressure (Figure 1F), urine protein/creatinine ratios (Figure 1G) and serum sFlt‐1 (Figure 1H). Moreover, renal damage including glomerular capillary endotheliosis and glomerular erythropenia (Figure 1I) and deterioration of development in terms of placental diameter and thickness, biparietal diameter, crown‐rump length and foetal heart rate (Figure 1J) were observed in the knockdown mice. Blockage of CD147 using a functional blocking antibody did not affect embryo implantation in mice (Figure S3).

**FIGURE 1:**
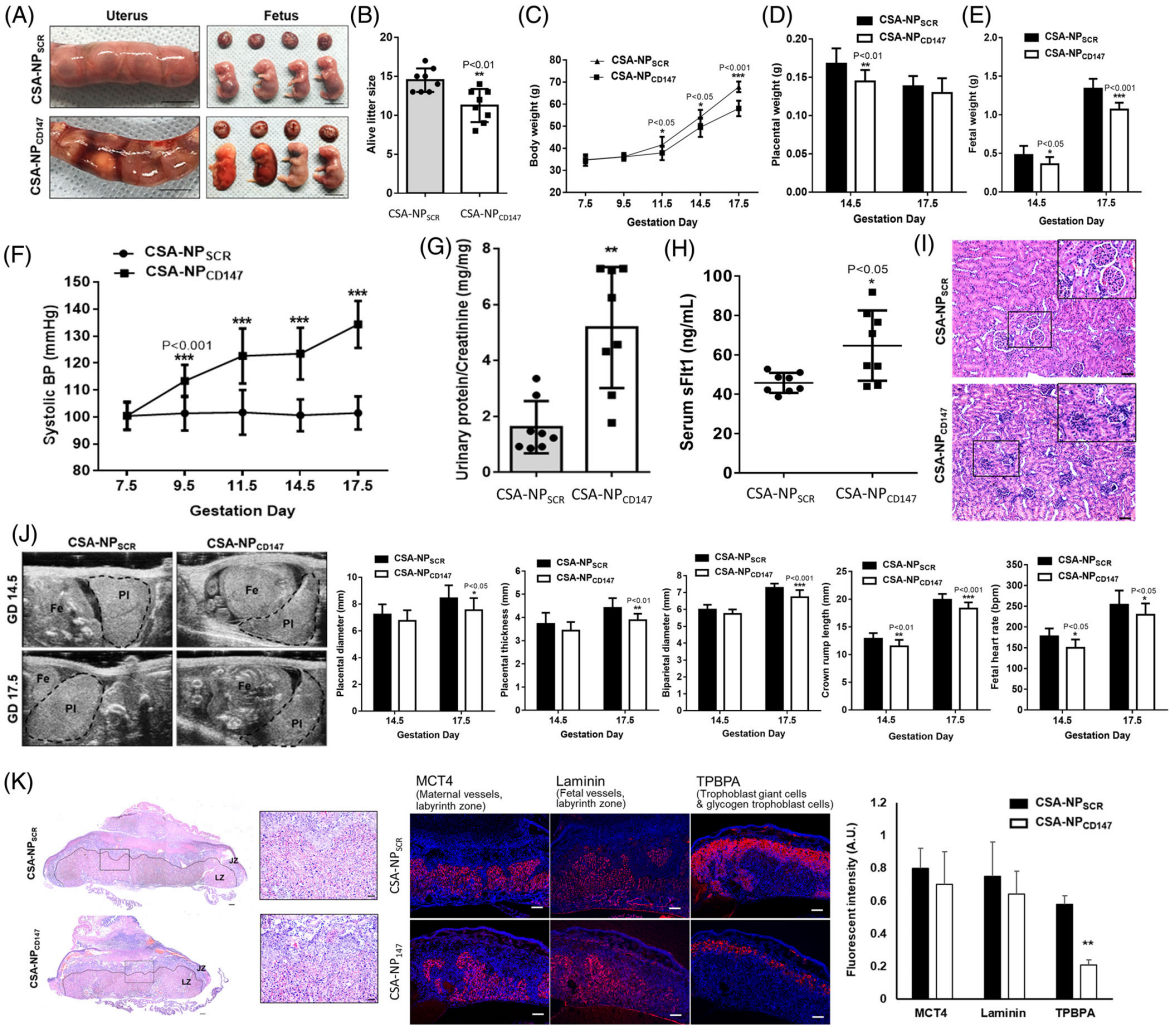
Placental‐specific CD147 knockdown induced preeclampsia (PE)‐like symptoms in the mouse model. (A) Representative images of gravid uteri, foetuses and placentas from CSA‐NP_SCR‐_ and CSA‐NPCD147‐treated mice on GD17.5 showing placental haemorrhage (scale bar = 1 cm). (B) Alive litter size from CSA‐NP_SCR‐_ and CSA‐NP_CD147_‐treated mice on GD17.5 (N = 8 litters). (C) Bodyweight from pregnant mice treated with CSA‐NP_SCR_ and CSA‐NP_CD147_ at GD7.5, 9.5, 11.5, 14.5 and 17.5 (N = 8). (D and E) Weight of placentas and foetuses collected at GD14.5 and 17.5 from CSA‐NP_SCR‐_ and CSA‐NP_CD147‐_treated pregnant mice (N = 5 litters). (F) Systolic blood pressure in CSA‐NP_SCR‐_ and CSA‐NP_CD147_‐treated pregnant mice at GD7.5, 9.5, 11.5, 14.5 and 17.5 (N = 8). (G) Urine protein/creatinine ratios and (H) serum sFlt‐1 levels of CSA‐NP_SCR_ and CSA‐NP_CD147_ group mice at GD17.5 (N = 8). (I) Renal histology in hematoxylin and eosin (HE)‐stained CSA‐NP_SCR‐_ and CSA‐NP_CD147_‐treated pregnant mice at GD17.5. Scale bar = 200 μm. (J) Evaluation of placental‐specific CD147 knockdown mouse embryos using an ultrasound imaging system. Representative images of embryos from CSA‐NP_SCR‐_ and CSA‐NP_CD147‐_treated pregnant mice at GD14.5 and 17.5 (Pl, placenta; Fe, foetus). Placental diameter, placental thickness, biparietal diameter, crown‐rump length and foetal heart rate were measured from different groups at GD14.5 and 17.5. (K) Placental histology in HE‐stained and placental expression of monocarboxylate transporter 4 (MCT4), laminin and trophoblast‐specific protein α (TPBPA) from CSA‐NP_SCR‐_ and CSA‐NP_CD147‐_treated pregnant mice at GD17.5. Left: Scale bar = 600 μm; Right: Scale bar = 100 μm. All data are expressed as the mean ± standard deviation, N = 8, * *P* < .05, ** *P* < .01 and *** *P* < .001. CSA‐NP_CD147_, plCSA‐BP‐modified CD147 Morpholino CD147‐Mor nanoparticles; CSA‐NP_SCR_, plCSA‐BP‐modified CD147‐mispair scramble morpholino nanoparticles; JZ, junctional zone; LZ, labyrinth zone

Dysregulated trophoblast differentiation contributes to placental dysfunction and PE.^2^ The trophoblastic CD147 knockdown mice exhibited a reduction in the area of the labyrinth and the number of trophoblast‐specific protein α (TPBPA)^+^ invasive trophoblast giant cells in the placenta (Figure 1K), suggesting defective trophoblast differentiation. Ablation of TPBPA^+^ cells in mouse placenta is associated with defective remodelling of the maternal spiral arteries.^7^ Indeed, CD147 has been proposed to regulate trophoblast differentiation in humans.^3^, ^4^ Analysis of single‐cell databases of the human cell landscape and human early maternal–foetal interface revealed high expression of CD147 in the cytotrophoblast (CT) and EVCT (Figure S4). Taken together, we hypothesised that CD147 regulated placental development in humans by modulating EVCT differentiation and functions. To test this hypothesis, we established human trophoblast stem cells (TSCs) from primary CT (Figure 2A) and trophoblast organoids from early placental villi (Figure 2B). Both models possessed molecular features of CT and functional features to differentiate into both EVCT and syncytiotrophoblast (ST) (Figure 2A,B). Our data showed that CD147 regulated the differentiation of EVCT but not ST cells in vitro (Figure 2A,B), consistent with the phenotypes of trophoblastic CD147 knockdown mice.

**FIGURE 2:**
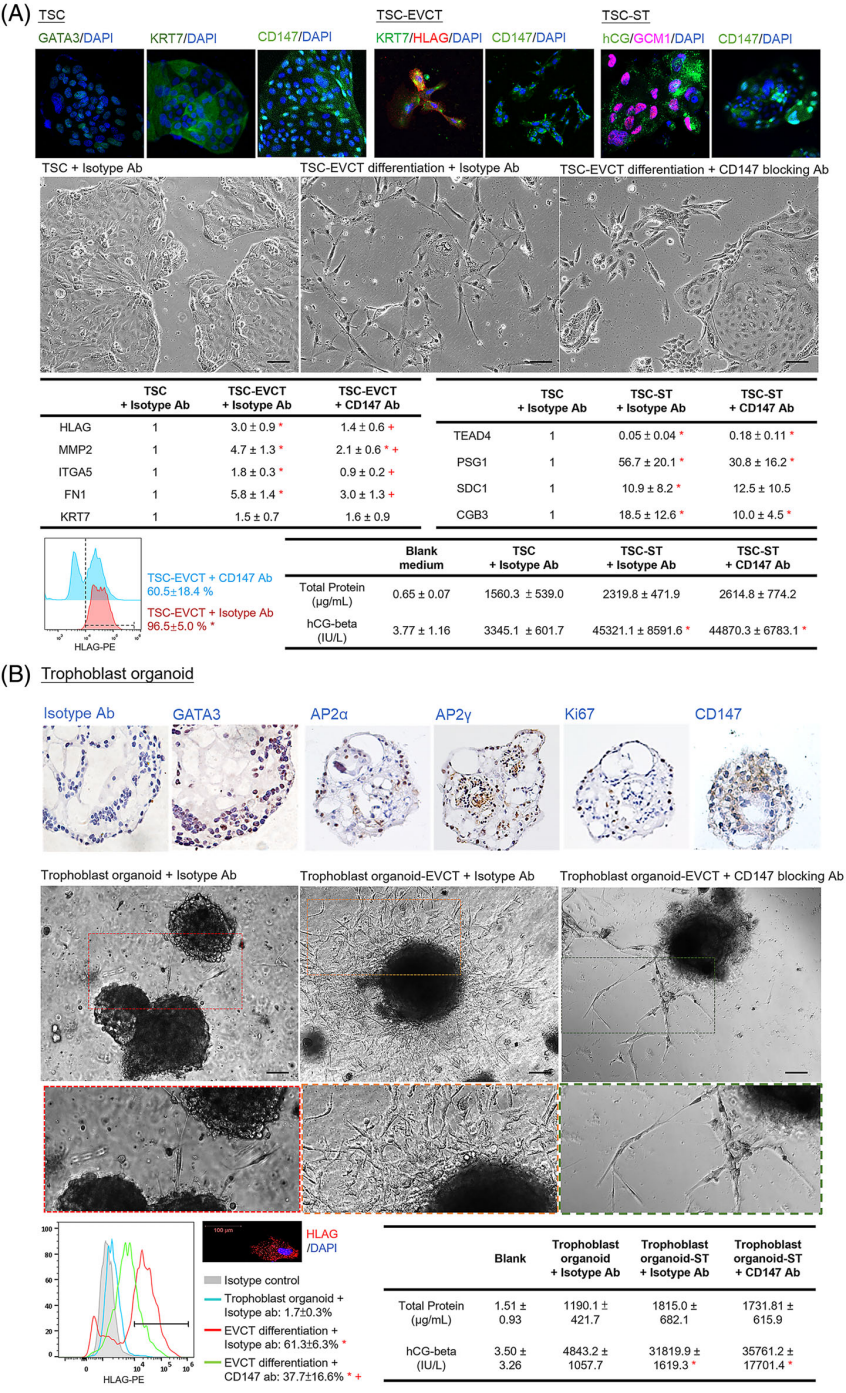
CD147 regulated extravillous trophoblast (EVCT) differentiation from Trophoblast stem cells (TSCs) and trophoblast organoids. (A) TSCs (GATA3^+^ KRT7^+^) were established from human cytotrophoblasts and were induced to differentiate into HLAG^+^ KRT7^+^ EVCT (TSC‐EVCT) and hCG^+^ GCM1^+^ ST (TSC‐ST). EVCT and ST differentiation of TSCs induced by differentiation medium and treated with CD147 blocking or isotype antibody (N = 4). (B) Trophoblast organoids (GATA3^+^ AP2α^+^ AP2α^+^) were established from human placental villi. The organoids were induced to differentiate into EVCT (trophoblast organoid‐EVCT) and ST (trophoblast organoid‐ST) cells. EVCT and ST differentiation of trophoblast organoids induced by differentiation medium and treated with CD147 blocking or isotype antibody (N = 4). All the data are expressed as the mean ± standard deviation. * *P* < .05 vs control. + *P* < .05 vs. TSC‐EVCT/TSC‐ST group. Scale bar = 100 μm. TSC‐EVCT, TSCs differentiate into EVCT; TSC‐ST, TSCs differentiate into ST; trophoblast organoid‐EVCT, trophoblast organoids differentiate into EVCT; Trophoblast organoid‐ST, trophoblast organoids differentiate into ST

Vascular remodelling of the spiral arteries by EVCT transforms the spiral arteries into low‐resistance and high‐flow vessels crucial for providing sufficient blood supply to the foetal–maternal interface in early pregnancy. Failure in vascular remodelling would give rise to placenta‐associated complications such as PE.^1^ We confirmed the expression of CD147 on primary EVCT and the EVCT‐like cell line JEG‐3 (Figure S1B). Suppression of CD147 functions by functional blocking antibody^8^ (Table S1) and siRNA (Figure S5) or stimulation of CD147 by ligation antibody^9^ (Table S1) showed that trophoblastic CD147 regulated characteristic features of the vascular remodelling process in early human pregnancy, including EVCT invasiveness and MMP2 expression/activity (Figure 3A,B), integration of EVCT into the endothelial network (Figure 3C), and endothelial cell angiogenesis (Figure 3D) and permeability (Figure 3E).

**FIGURE 3:**
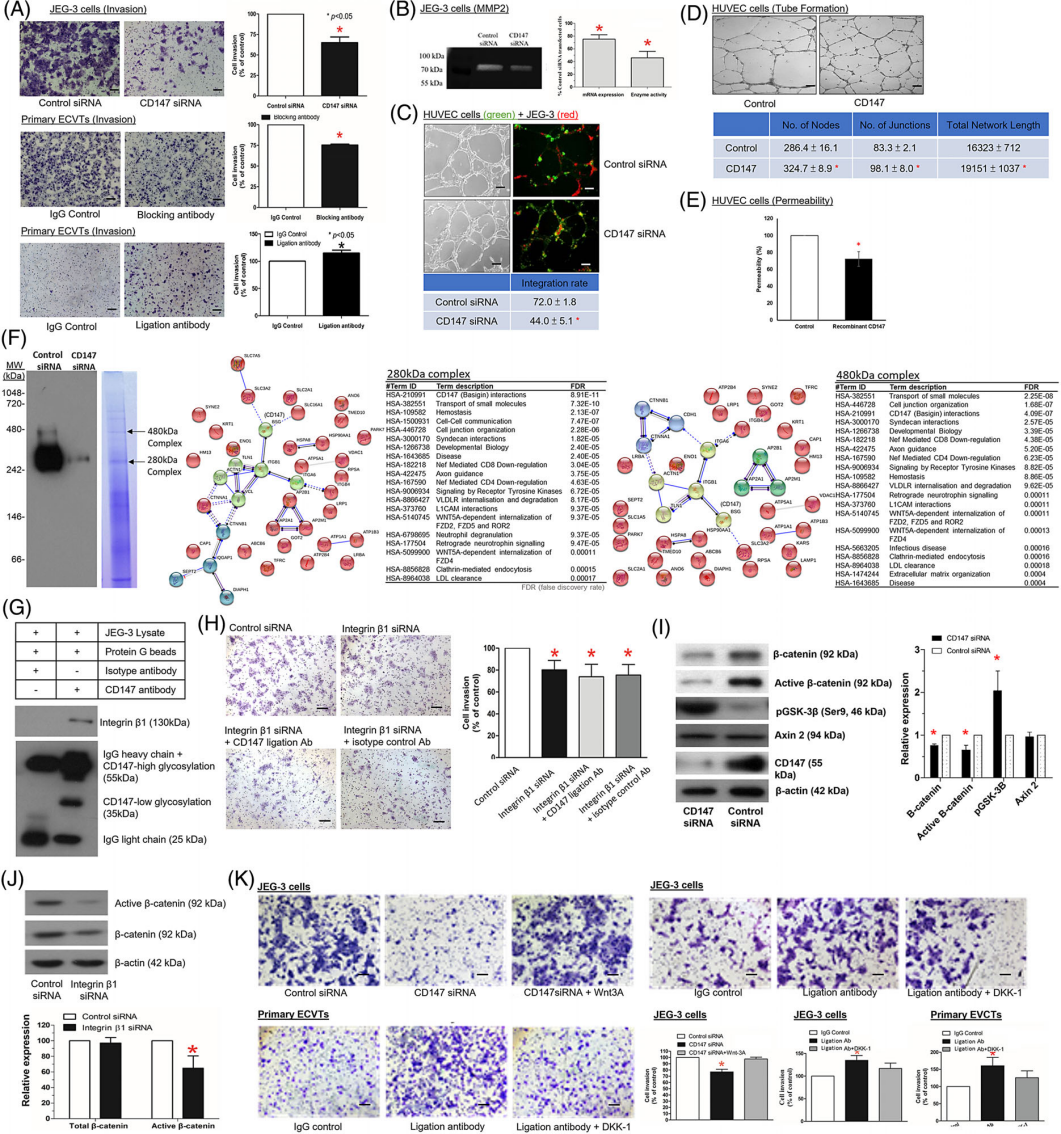
CD147 regulated the spiral artery remodelling functions of extravillous trophoblast (EVCT) through CD147‐integrin β1 membrane complexes and Wnt/β‐catenin signalling. (A) Transwell invasion assay of control or CD147 siRNA‐suppressed JEG‐3 cells (3 × 10^5^) (N = 5), primary EVCTs (3 × 10^6^) treated with isotype control, CD147 functional blocking Ab (N = 4) and primary EVCTs (1 × 10^6^) treated with CD147 stimulatory ligation Ab (N = 4). (B) MMP2 mRNA expression (N = 15) and activity (N = 5) of control and CD147‐suppressed JEG‐3 cells. (C) Green fluorescence‐labelled endothelial cells (HUVECs) were cocultured with red fluorescence‐labelled control or CD147‐suppressed JEG‐3 cells. EVCT integration into the endothelial tubing was then quantified by confocal microscopy (N = 5). (D) Tube formation assay and (E) permeability assay of HUVECs treated with recombinant CD147 (N = 5). (F) Membrane protein complexes were resolved by blue native gel electrophoresis (N = 3). Western blot analysis of the blue gel identified CD147 in two complexes corresponding to 280 and 480 kDa, and both complexes were downregulated after siRNA suppression (N = 5). Protein identification of the CD147 membrane complex by liquid chromatography‐tandem mass spectrometry. Protein interactions were analysed using the STRING and Reactome pathway databases. Minimum required interaction score confidence = 0.95. (G) Protein interaction between CD147 and integrin β1 in the immunoprecipitation experiment (N = 5). (H) Integrin β1 suppression abolished the stimulatory effect of CD147 ligation Ab in JEG‐3 cell invasion (N = 5). (I) CD147 regulated EVCT invasiveness via Wnt/β‐catenin signaling. Total β‐catenin, active β‐catenin, p‐GSK3β (Ser9), Axin2, CD147 and β‐actin protein expression in control or CD147 siRNA‐suppressed JEG‐3 cells was determined by western blotting (N = 10). (J) β‐catenin and active β‐catenin expression in control or integrin β1‐suppressed JEG‐3 cells was determined by western blotting (N = 10). (K) Invasion of control or CD147 siRNA‐suppressed JEG‐3 cells after cotreatment with Wnt3A (N = 5) and invasion of JEG‐3 cells (N = 5) and primary EVCTs (N = 4) treated with CD147 stimulatory ligation ab and Wnt inhibitor DKK‐1. Scale bar = 100 μm. All the data are expressed as the mean ± SD. * *P* < .05

CD147 has an unusual transmembrane domain characterised by a single‐charged glutamic acid in the hydrophobic transmembrane region, which promotes protein–protein interactions.^4^ CD147 is a component of two receptor complexes (Figure 3F; Table S2) on the plasma membrane of human EVCT. The CD147‐integrin β1 interaction within the complex was confirmed by protein interaction analysis (Figure 3F), coimmunoprecipitation (Figure 3G) and their specific expression in EVCT (Figures S1C and S4). The interaction of CD147 with integrin‐β1 (Figure 3H; Figure S5) and with Wnt/β‐catenin signalling (Figure 3I‐K) mediates the activities of CD147 on EVCT invasion.

CD147 has been applied as a predictive marker or therapeutic target of cancer.^10^ Based on the roles of CD147 in regulating placentation in vivo and EVCT functions in vitro, we speculated that deficiency of CD147 might contribute to the pathophysiology of early‐onset PE. This was supported by our clinical data, which revealed downregulation of placental CD147 (Figure 4A) and serum soluble CD147 (Figure 4B) in early pregnant women who developed PE in late pregnancy when compared to normotensive controls (Table S3). Angiogenic factors including PlGF or sFlt‐1:PlGF ratio, and novel markers such as cell‐free RNA have been investigated as predictive biomarkers for PE during early pregnancy. Given the clinical heterogeneity of PE, there is a need to further develop an early prediction biomarker panel that reflects different pathophysiological processes of PE. The clinical significance of CD147 as a test for early prediction or prognostic marker of PE when combined with other established biomarkers is worth further investigation in larger clinical trials.

**FIGURE 4:**
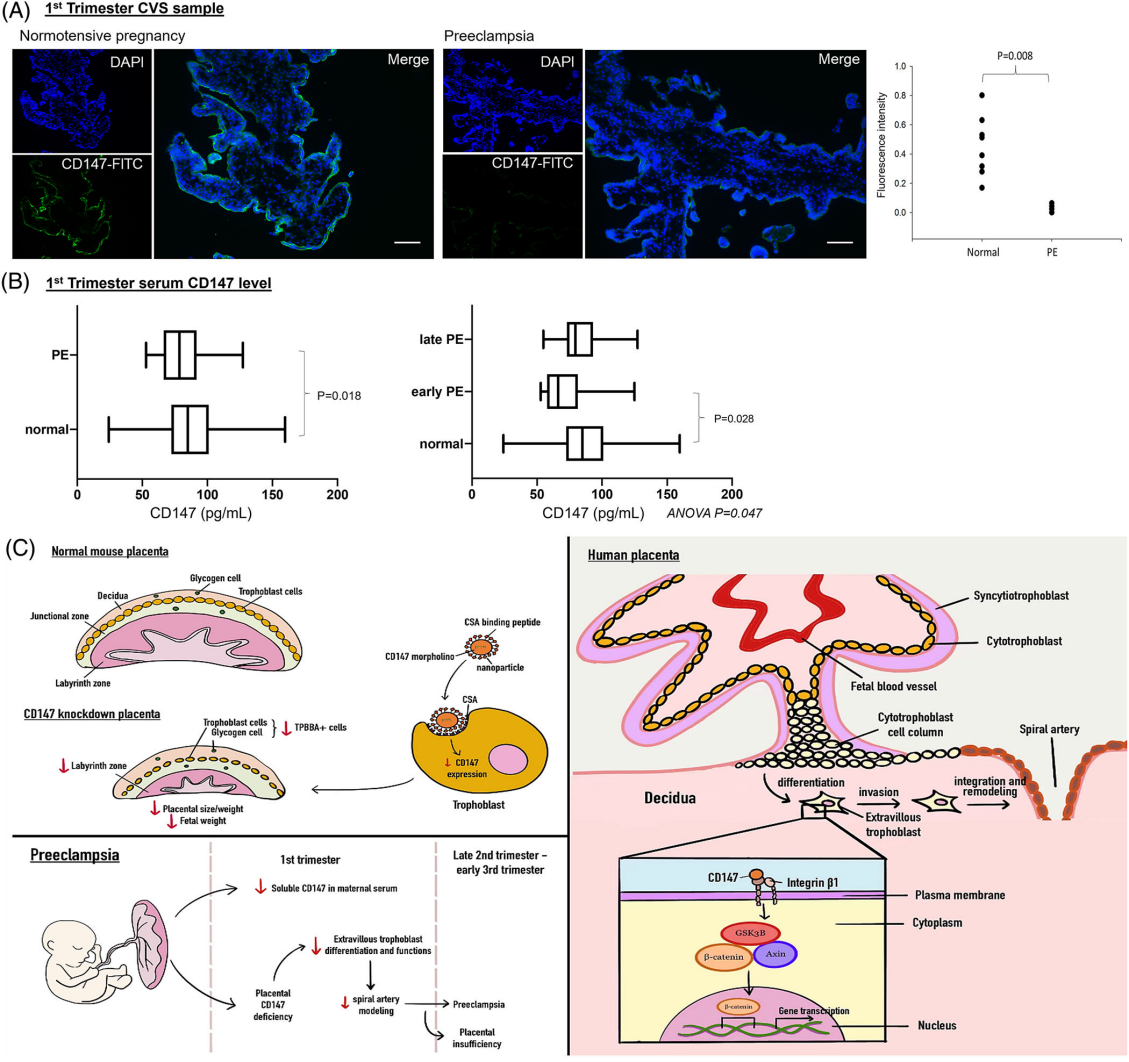
CD147 expression was reduced in first trimester placental villi and maternal serum from early pregnant women who developed preeclampsia (PE) at term. (A) Expression of CD147 in early pregnancy of normotensive and PE chorionic villous samples (normotensive N = 8, PE N = 6). Quantitative analysis was performed using Image‐Pro Plus software. (B) First trimester serum CD147 levels of the normotensive and PE pregnant women were measured by ELISA (normotensive N = 52, PE N = 26, early‐onset PE = 8, late‐onset PE N = 18). Data are expressed as the median (Box: 25th and 75th percentile; Error bar: range). (C) A summary of dysregulation of the CD147 complex confers defective placental development and its association with preeclampsia. In pregnant mice, placenta‐specific suppression of CD147 led to preeclampsia‐like phenotypes and defective placental development. In vitro functional assays showed that CD147 mediates the differentiation and spiral artery remodelling activities of EVCT. In preeclampsia pregnancy, CD147 expression in first trimester chorionic villous and serum samples was reduced when compared with that in normal pregnancy

In summary, by studying the roles and mechanisms of CD147 in trophoblast differentiation and functions, we provide novel evidence that defective trophoblastic CD147 expression is a placental cause of PE (Figure 4C). Specifically, the defect affects the differentiation and vascular remodelling activities of EVCT and thereby disrupts normal placental development. Clinically, the results of this study indicate a possible research direction for the use of CD147 for the early prediction of PE.

## CONFLICT OF INTEREST

The authors declare no conflict of interest.

## Supporting information



Supporting information.
