# Supplementary material for: Dysregulation of the CD147 complex confers defective placental development: A pathogenesis of early‐onset preeclampsia
Source: Clin Transl Med. 2022 Jun 2;12(6):e826. doi: 10.1002/ctm2.826 (PMC9162301; doi:10.1002/ctm2.826)
Supplement: Supplementary file 1 — Supporting information. [file CTM2-12-e826-s001.docx]

**Supplementary Materials**

**Dysregulation of CD147 complex confers defective placental development: a pathogenesis of early-onset preeclampsia.**

Cheuk-Lun Lee^1,2#*^, Zhilong Chen^3,4#^, Qingqing Zhang^1,2#^, Yue Guo^2^, Vivian W.Y. Ng^2^, Baozhen Zhang^3,5^, Kunfeng Bai^1,2^, Degong Ruan^6^, Anita S.Y. Kan^2^, Ka-Wang Cheung^2^, Annisa Shui Lam Mak^7^, William S.B. Yeung^1^, Rui Su^3,4^, Qing Yang^4^, Min Chen^8^, Mei-Rong Du^9^, Zhang Jian^3^, Xiujun Fan^3*^, Philip C.N. Chiu^1,2*^

^1^The University of Hong Kong Shenzhen Key Laboratory of Fertility Regulation, The University of Hong Kong-Shenzhen Hospital. ^2^Department of Obstetrics and Gynaecology, LKS Faculty of Medicine, The University of Hong Kong. ^3^Center for Energy Metabolism and Reproduction, Shenzhen Institute of Advanced Technology, Chinese Academy Sciences. ^4^College of Veterinary Medicine, Hunan Agricultural University, Changsha 410128, China. ^5^University of Chinese Academy of Sciences, Beijing, 100049, China. ^6^Stem Cell & Regenerative Medicine Consortium, School of Biomedical Sciences, The University of Hong Kong. ^7^Department of Obstetrics and Gynecology, Queen Elizabeth Hospital, Hong Kong. ^8^Department of Fetal Medicine and Prenatal Diagnosis, The Third Affiliated Hospital of Guangzhou Medical University, Guangzhou, China. ^9^NHC Key Lab of Reproduction Regulation (Shanghai Institute of Planned Parenthood Research), Hospital of Obstetrics and Gynecology, Fudan University Shanghai Medical College, Shanghai, China.

* Dr. Philip C.N. Chiu, Tel: 852-39176996, Fax: 852-28161947, Email: pchiucn@hku.hk; Dr. Cheuk-Lun Lee, Tel: 852-39179388, Fax: 852-28161947, Email: kcllee@hku.hk; Dr. Xiujun Fan, Tel: 075586392360, Fax: 86-755-86585222, Email: xiujun.fan@gmail.com

**Introduction**

Preeclampsia (PE) is a gestational complication and the top cause of prenatal mortality and morbidity. The etiology of preeclampsia is associated with defective trophoblast differentiation and functions causing abnormal placental development and maternal-fetal exchange defects. CD147, also known as basigin (BSG), is a key component of protein complexes on human trophoblast which has been linked to the physiology and pathology of several reproductive processes. This study investigates the roles of trophoblastic CD147 in placental development and its association with preeclampsia.

**Methods**

**Ethics approval and consent to participate**

The involvement of human subjects in this study was approved by the Institutional Review Board of The University of Hong Kong/Hospital Authority Hong Kong West Cluster (UW13-083 & UW21-184). The animal experimental protocols were approved by the Animal Ethics Committee of the Shenzhen Institutes of Advanced Technology, Chinese Academy of Sciences.

**Collection of early pregnancy chorionic villus and maternal serum**

The criteria for defining PE during pregnancy include (1) blood pressure (BP) of 140/90 mm Hg or greater after 20 weeks of gestation, (2) proteinuria of 300 mg/L or greater, and (3) without previous hypertensive or renal disease history.

Chorionic villus sampling (CVS) and maternal serum samples, which surplus to diagnostic need, were obtained from pregnant women undergoing prenatal diagnosis in early pregnancy (11–13+6 weeks of gestation). 198 CVS samples and 790 maternal blood samples were collected from the prenatal diagnosis clinics at Tsan Yuk Hospital and Queen Elizabeth Hospital. As there was no reliable marker for the prediction of PE in early pregnancy, these samples were stored until delivery when the PE status was known. Six of the CVS samples and 26 of the maternal serum samples (early-onset PE=8, late-onset PE=18) developed PE at delivery. The incidence of PE in the cohort was ~3% (6/198 & 26/790), similar to the reported incidence in Hong Kong^1^.

**Trophoblast isolation and culture**

Extravillous trophoblast (EVCT) and Cytotrophoblast (CT) were isolated from human first-trimester placental villi as described^2,3^ from women undergoing surgical termination of pregnancies due to psychosocial reasons. They were immuno-magnetic isolated based on the expression of human leukocyte antigen G, HLA-G (Clone MEM-G/9, Abcam) and CD49f (Clone GoH3, Miltenyi Biotec), respectively as described^3-5^. Human choriocarcinoma cell line JEG-3 expressing EVCT marker HLA-G^6,7^ was obtained from American Type Culture Collection (Manassas, USA). Human umbilical vein endothelial cells (HUVECs) were obtained from Lonza (Basel, Switzerland).

Primary EVCT and JEG-3 cells were cultured in Dulbecco's Modified Eagle Medium (DMEM)/F-12 (Thermo Fisher Scientific, Waltham, MA, USA). HUVEC cells were cultured in HAM/F-12 (Sigma-Aldrich) medium supplemented with 1% endothelial cell growth supplement (Sigma-Aldrich), 0.1% heparin (Sigma-Aldrich). The media were supplemented with 10% FBS and 1% penicillin and streptomycin, and the cells were cultured in a humidified atmosphere of 5% CO_2_ in air at 37°C.

**Human trophoblast stem cells (TSC) and trophoblast organoid culture**

By activation of Wingless/Integrated (Wnt) and EGF, inhibition of TGF-β, histone deacetylase, and Rho-associated protein kinase, human TSC were derived from human primary CT^3,8^. In this study, human TSC were established from CT of human placenta at early pregnancy in TSC medium (DMEM/F12 supplemented with 0.1mM 2-mercaptoethanol, 0.2% FBS, 0.5% penicillin-streptomycin, 0.3% BSA, 1% ITS-X supplement, 1.5 mg/mL L-ascorbic acid, 50 ng/mL EGF, 2 µM CHIR99021, 0.5 mM A83-01, 1 µM SB431542, 0.8 µM valproic Acid and 5 µM Y27632).

Human trophoblast organoids were derived from primary first trimester placenta tissue as described^9^ in organoid medium (DMEM/F12 supplemented with 0.5% penicillin-streptomycin, B27 supplement minus vitamin A, N2 supplement, 2 mM L-glutamine, 1.25 mM N-acetyl-L-cysteine, 500 ng/mL rspondin-1, 50 ng/mL EGF, 100 ng/mL FGF-2, 50 ng/mL HGF, 2 µM CHIR99021, 5 µM Y27632, 500 nM A83-01, 2.5 µM PGE2 and 100 µg/mL Primocin). The organoids were anatomically and functionally resembling the villous placenta ^9^, composed of CT and ST, and were capable of differentiating into EVCT.

**Immunohistochemistry and immunofluorescence staining**

Human and mouse placental tissues were fixed in 4% formalin overnight, rinsed in PBS, and transferred to 70% ethanol before standard processing to obtain paraffin-embedded sections (5 µm). Immunohistochemistry was performed on the sections after permeabilization in buffer (eBioscience™ Permeabilization Buffer, Thermo Fisher Scientific) using primary antibodies listed in Supplemental Table ST1, followed by the corresponding biotinylated secondary antibodies. Immunodetection was performed by VECTASTAIN Elite ABC kit (PK6100, Vector Laboratories) according to the manufacturer’s instructions using 3,3′-diaminobenzidine tetrahydrochloride (Dako) as chromagen. The slides were counterstained with hematoxylin and mounted in permount^TM^ medium (Thermo Fisher Scientific) and observed under a light microscope (Zeiss). An H-score based on the percentage of cells at each staining intensity was determined using the formula: [1×(% cells 1^+^) + 2×(% cells 2^+^) + 3×(% cells 3^+^)], where 1^+^, 2^+^ and 3^+^ referred to different staining intensities. Immunofluorescence staining was performed with the antibodies listed in Supplemental Table ST1. Nuclei were stained with DAPI. The slides were observed under Zeiss Laser scanning confocal microscopes (LSM 700, 880 & 900, Faculty Core Facility, The University of Hong Kong) and were quantified by the Image-Pro Plus software (Media Cybernetics, Inc., MD, USA).

**Enzyme-linked immunosorbent assay (ELISA)**

The human serum level of CD147 and mouse serum level of sFlt-1 were measured by ELISA (CD147, Abcam, ab221437; sFlt-1, Boster, EK0589) according to the manufacturer's protocols. In brief, serum samples were added to the capture antibody-coated wells of a 96-well microplate and were incubated overnight at 4°C. After washing with the provided washing buffers, matched biotin-labeled detection antibody was then added to the wells for incubation. Horseradish peroxidase and 3,3’,5,5’-tetramethylbenzidine were used for detection. The reaction was stopped by the addition of 2 M sulfuric acid and the absorbance at 450 nm was measured using a microtiter plate reader. A standard curve was obtained by serial dilutions that covered the entire detection range of the assay. The sensitivities of the CD147 and sFlt-1 ELISAs were 7.8 and 156 pg/mL, respectively.

**Western blotting**

Cells or tissues were lysed with the Cytobluster^TM^ protein extraction reagent (Novagen) or the Membrane Protein Extraction Reagent (Thermo Fisher Scientific) with a protease inhibitor cocktail (Thermo Fisher Scientific). The concentration of protein was measured using the Pierce™ BCA Protein Assay Kit (Thermo Fisher Scientific). Equal amounts of protein were separated by sodium dodecyl sulfate-polyacrylamide gel electrophoresis and transferred to a polyvinylidene fluoride membrane. Western blot analyses were carried out using antibodies listed in Supplemental Table ST1 followed by the corresponding biotinylated secondary antibodies. Protein bands were quantified by densitometry using the Quantity One software (Bio-Rad, Hercules, CA, USA).

**RNA isolation and quantitative real-time PCR (qPCR)**

Total RNA was extracted using the QuickPrep RNA extraction kit (GE Healthcare), and reverse transcribed using the TaqMan Reverse Transcription Reagent (Applied Biosystems). qPCR was conducted in a QuantStudio 5 Real-Time PCR System (Applied Biosystems) using TaqMan qPCR assay probes. The probes consisted of the pan-trophoblast marker: cytokeratin-7 (KRT7, Hs00559840_m1); TSC marker: GATA binding protein 3 (GATA3, Hs00231122_m1), TEA domain transcription factor 4 (TEAD4, Hs01125042_mH); EVCT markers: human leukocyte antigen G (HLAG, Hs00365950_g1), matrix metallopeptidase 2 (MMP2, Hs01548727_m1), integrin subunit alpha 5 (ITGA5, Hs01547673_m1), fibronectin 1 (FN1, Hs01549976_m1); ST markers: pregnancy-specific beta-1-glycoprotein 1 (PSG1, Hs04235345_s1), syndecan 1 (SDC1, Hs00174579_m1) and chorionic gonadotropin subunit beta 3 (CGB3, Hs00361224_gH). 18S rRNA (Hs99999901_s1) was used as the internal control. The reactions were performed in triplicate. The Threshold Cycle (CT) method (2−^△△CT^ method) was applied to calculate the relative gene expression.

**Non-invasive placenta-specific CD147 suppression mouse model**

The chondroitin sulfate A binding peptide (plCSA-BP)-coated nanoparticles are developed for placenta-specific drug delivery (https://www.jove.com/cn/t/58219/comprehensive-evaluation-effectiveness-safety-placenta-targeted-drug?status=a60225k)^10,11^. As systematic CD147 gene knockout leads to perinatal lethality^12^, the plCSA-BP nanoparticle method was used to suppress CD147 expression specifically in mouse placenta. CD147 morpholino (TTTGAGCACTGTGGACTCACATGT), green-fluorescence labeled CD147 morpholino and CD147-mispair scramble morpholino (TTTCACCACTCTGCACTCACATCT) were purchased from 4A Biotech (Beijing, China). The plCSA-BP (EDVKDINFDTKEKFLAGCLIVSFHEGKC) was purchased from China Peptides Co., Ltd. (Shanghai, China). plCSA-BP-coated nanoparticles loaded with CD147 morpholino (CSA-NP_CD147_), green-fluorescence modified CD147 morpholino (CSA-NP_CD147-GFP_) and CD147-mispair scramble morpholino (CSA-NP_SCR_) were synthesized as described^10^. The diameter and zeta potential of the nanoparticles were measured by a Zetasizer Nano 2000 (Malvern, England). To measure cellular uptake of the nanoparticles, JEG-3 cells were incubated with the nanoparticles (10 µM) for 1 h at 4°C. The toxic effects of the nanoparticles were studied by the Cell Counting Kit-8 (Sigma-Aldrich). The efficiency of the nanoparticles to suppress CD147 was accessed by western blot analysis.

Six-week-old CD1 mice (Beijing Vital River Laboratory Animal Technology Co., Ltd., Beijing, China) were housed in a pathogen-free animal room with a 14h light/10h dark cycle. Nanoparticles (10 µM) were injected through the tail vein to pregnant mice on gestational day (GD) 5.5, 7.5, 9.5 and 11.5 (Vaginal plug = GD0.5). The pregnancy mice at GD14.5 and 17.5 were anesthetized with 5% isoflurane at 1L of O_2_/min and maintained under 3% isoflurane at 1L of O_2_/min. Mouse embryo development was monitored by a VEVO2100 imaging system (VisualSonics, Toronto, Canada)^10^, and blood pressure was measured on GD7.5, 9.5, 11.5, 14.5 and 17.5 by a non-invasive computerized tail-cuff method with a BP-2000 Visitech System (Visitech systems Inc, Apex, NC)^10^. Placental and fetal weight on GD15.5 and 17.5 were measured. Morphology of the placenta and kidney was accessed by hematoxylin-eosin staining. Urinary albumin and creatinine at GD17.5 were measured by commercial kits (E024, Beijing5 HYCX, China; C011-2, Nanjing Jiancheng Bioengineering Institute, China).

**Trophoblast differentiation analysis**

To induce differentiation of TSC into EVCT^3,8^, TSC were cultured in a 12 well plate pre-coated with 1% Matrigel® Matrix (Corning, USA) in 1 mL of EVCT differentiation medium (DMEM/F12 supplemented with 0.1 M M2-mercaptoethanol, 0.5% penicillin-streptomycin, 0.3% BSA, 1% ITS-X supplement, 100 ng/mL NRG1, 7.5 µM A83-01, 2.5 µM Y27632, 4% KnockOut Serum Replacement and 2% Matrigel). The medium was replaced with the EVCT medium without NRG1 and in 0.5% Matrigel on day 3 and with EVCT medium without NRG-1/KnockOut Serum Replacement and in 0.5% Matrigel on day 6. The cells were harvested on day 8 for EVCT differentiation measurement. For syncytiotrophoblast (ST) differentiation, TSCs (5×10^4^) were treated with CD147 functional blocking/isotype antibodies in a 12-well plate pre-coated with 2.5 mg/ml Col IV in 1 mL of ST medium (DMEM/F12 supplemented with 0.1mM 2-mercaptoethanol, 0.5% penicillin-streptomycin, 0.3% BSA, 1% ITS-X supplement, 2.5 mM Y27632, 2 mM forskolin and 4% KnockOut Serum Replacement). The medium was replaced on day 3 and the cells were harvested on day 6 for ST differentiation measurement. TSC (1.5×10^4^) were treated with anti-CD147 functional blocking antibody (5µg/mL, ab49493/ab232967, Abcam) ^13^ or mouse IgG isotype control antibody (12-371, Merck Millipore). They were then allowed to differentiate into EVCT/ST using the differentiation medium as mentioned above.

The organoid model allows morphogenetic study of EVCT differentiation. It retains the normal placental villous structure with allows differentiation of EVCT that invade and digest the surrounding Matrigel^9^. Trophoblast organoids were differentiated into EVCT and ST using the same protocols as for TSCs. For measuring EVCT differentiation, the organoids in Matrigel were dissolved in cell recovery solution and the differentiated EVCTs were obtained by filtering through a 40µm cell strainer.

The expressions of EVCT, ST, TSC markers were measured by qPCR assays. The expression of HLA-G was measured by flow cytometry (Cytoflex, Beckman Coulter, IN, USA). The data were analysed by the FlowJo software (Tree Star Inc., Ashland, USA). The total protein and hCG concentrations of the conditioned media were measured by the BCA assay kit (Thermo scientific) and an immunoassay analyser (The Architect, i1000SR), respectively.

**EVCT assays**

JEG-3 cells were transfected with siRNA (72.5 pmol/mL) against human CD147 (s2098 and s2099, ratio 1:1), integrin β1 (s7574 and s7575, ratio 1:1), or negative control siRNA (AM4611 and AM4613, ratio 1:1) using the Lipofectamine 2000 transfection reagent in Opti-MEM Media according to the manufacturer's protocol (Thermo Fisher Scientific). Primary EVCT (1×10^6^) were treated with anti-CD147 functional blocking antibody (5 µg/mL, ab49493, Abcam)^13^, stimulatory ligation antibody (5 µg/mL, ab119114, Abcam)^14^ and mouse IgG isotype control antibody (Merck Millipore) for functional assays. The CD147 stimulatory ligation antibody recognized a unique epitope and stimulated CD147-mediated biological function in T-cell^14^.

Transwell invasion (354480, Corning, USA) and migration assays (CytoSelect™ 24-Well Cell Migration Assay, Cell Biolabs, USA) were conducted as described^2,15^. The invasiveness of primary EVCT or JEG-3 cells was quantified by measuring the absorbance at 595 nm and calculated by the equation: Relative invasion/migration (%) = (Absorbance of treatment / absorbance of control) × 100%. MMP-2 activities were measured by gelatin zymography as described^16,17^.

Trophoblast integration was measured by an *in vitro* EVCT-endothelial cells co-culture assay as described^4,18^. Green fluorescence-labeled (CellTracker™ Green CMFDA Dye, Thermo Fisher Scientific) human umbilical vein endothelial cells (HUVEC, 5x10^4^ cells, Lonza, Basel, Switzerland) were allowed to form an endothelial tube network on Matrigel-coated plates for 4 h. After that, red fluorescence-labeled (CellTracker™ Red CMTPX Dye, Thermo Fisher Scientific) JEG-3 cells (0.5x10^5^) were added into the endothelial network for 14 h. Integration was quantified by confocal microscopy and calculated as Integration (%) = (EVCT red fluorescent area / HUVEC green fluorescent area) × 100%.

The viability of EVCT was assessed by a colorimetric assay (Cell Proliferation Kit II, Sigma-Aldrich) as described^5^. Viability (%) = (Absorbance of treatment - absorbance of blank / absorbance of control - absorbance of blank) × 100%.

**Endothelial cell tube formation assays**

The angiogenic function of endothelial cell was evaluated by the formation of HUVEC into a capillary tube-like network as described^4^ in the presence of recombinant CD147 (5 μg/mL, 972-EMN-050, R&D Systems) for 15 h. Angiogenesis parameters including the number of nodes, number of junctions and total tube network length were analysed using the angiogenesis plugin (National Institute of Health, USA) of the Image J software (Media cybernetics, Inc. Washington, USA). The permeability of HUVEC was measured by a vascular permeability assay kit (ECM640, Merck Millipore) and was calculated as Permeability (%) = (Absorbance of treatment - absorbance of blank / absorbance of control - absorbance of blank) × 100%.

**Identification of protein complexes by blue native gel electrophoresis and nano-liquid chromatography-tandem mass spectrometry**

The membrane protein complexes of EVCT were prepared by the NativePAGE^TM^ Sample Prep Kit (Thermo Fisher Scientific) according to the manufacturer’s protocol. The protein complexes were separated by blue native gel electrophoresis on 4-12% gradient polyacrylamide gels, and the CD147 complexes were identified by Western blot analysis using the anti-CD147 antibody. The composition of the CD147 complexes in the BNG gel bands were identified by nano-liquid chromatography-mass spectrometry as described^19^ with a nano-reversed phase C18 column connected to an LC/MS-MS-TripleTOF 5600 system (AB Sciex, Canada). The ProteinPilot Software (AB Sciex, Canada) was used to match peptide mass spectra with protein sequences in the UniProt (http://www.uniprot.org) using the *Homo Sapiens* transmembrane proteome subset. Peptides were identified based on a statistical confidence of 99% (Unused Score>2) sequence matching. Protein interactions of the top 40 proteins were identified by STRING (Search Tool for the Retrieval of Interacting Genes/Proteins, https://string-db.org) using Kmean clustering with a minimum required interaction score confidence of 0.95.

**Interaction between CD147 and integrin β1**

Co-expression of CD147 and integrin β1 on JEG-3 cells and EVCT were determined by immunofluorescence staining. Co-immunoprecipitation was conducted to confirm the physical interaction between CD147 and integrin β1. Briefly, 250 µg JEG-3 total cell lysate was incubated with monoclonal anti-CD147 (ab11572, Abcam) or isotype antibody overnight at 4^o^C. Protein-G PLUS-Agarose beads (GE Healthcare) were added to capture CD147 and its interacting partners. The beads were then washed, denatured and analysed by western blot using antibodies against CD147 (ab232967, Abcam) and integrin β1 (ab30394, Abcam). To study the biological function of the interaction, the expression of integrin β1 on JEG-3 was suppressed by siRNA. Effect of CD147 stimulation on invasion of integrin β1 suppressed JEG-3 cells was determined. The co-expression of integrin β1 and CD147 in the JEG-3 cells and primary EVCTs were measured by immunohistochemistry.

**Effect of CD147 on β-catenin signaling pathway in EVCT**

Effect of CD147 siRNA suppression on the expression of active β-catenin, total β-catenin, phosphorylated (Ser9) GSK3β and Axin2 in JEG-3 cells were detected by Western blot. Effect of Wnt activator Wnt-3a (200 ng/ml, 5036-WN/CF, R&D Systems, Minneapolis, USA) in CD147 suppressed JEG-3 cells and effect of inhibitor DKK-1 (1µg/ml, 5439-DK/CF, R&D Systems) in CD147 stimulated JEG-3 cells/EVCTs on cell invasion were determined.

**Protein expression pattern analysis**

Protein expression patterns of CD147 and integrin β1 in human tissues and the feto-maternal interface were determined using the single-cell analysis database of human cell landscape^20^ (https://db.cngb.org/HCL/) and the early maternal-fetal interface in humans^21^ (Supplemental Figure S4). The scripts of the bioinformatic analysis were uploaded to a Github page (https://github.com/JannisLee/Analysis_CD147).

**Statistical analysis**

All the experimental data were expressed as the mean ± SD (standard deviation) and was analysed by statistical software (SigmaPlot 11.0; Systat Software, Inc., USA and SigmaStat 2.03; Jandel Scientific, San Jose, CA, USA). Non-parametric analysis of variance on the rank test was used to identify group differences followed by Mann-Whitney U-test as the post-test. A probability value of <0.05 was considered statistically significant.

**Results**

**Placenta-targeted nanoparticles loaded with anti-CD147 morpholino antisense oligonucleotides induce PE-like phenotypes in pregnant mice**

CD147 is expressed on human and mouse placenta (Supplemental Figure S1A). To determine the effect of CD147 down-regulation on placental and fetal development, a placenta-specific CD147 knockdown mouse model was generated by plCSA-BP-coated nanoparticles. The nanoparticles that bound specifically to the trophoblast were loaded with anti-CD147 morpholino antisense oligonucleotides using the sonication method^22^ (Supplemental Figure S2A). CD147-mispair scramble morpholino was used as the control. The size distributions of the plCSA-BP-conjugated nanoparticles loaded with CD147-morpholino (CSA-NP_CD147_) and CD147-mispair scramble morpholino (CSA-NP_SCR_) were similar as determined by dynamic light scattering (Supplemental Figure S2B).

CD147 was highly expressed in the junctional zone and labyrinth zone of the mouse placenta (Supplemental Figure S1A). In vitro experiments demonstrated specific binding of the nanoparticles to mouse migratory trophoblast cells (MTB) cells, but not mouse fibroblasts (3T3-L1 cells) (Supplemental Figure S2C) with low cytotoxicity (Supplemental Figure S2D). The expression of CD147 protein in the mouse placenta was gradually increased as the pregnancy progressed and peaked at GD17.5 (Supplemental Figure S2E). Pregnant mice were injected intravenously with CSA-NP_CD147_ and CSA-NP_SCR_ on GD5.5, 7.5, 9.5 and 11.5 (Supplemental Figure S2F). The nanoparticles were specifically bound to and taken up by the trophoblast in the placenta (Supplemental Figure S2F). The CSA-NP_CD147_ significantly suppressed the expression of placental CD147 mRNA (by 37.5±4.7%) and protein (by 43.3±3.1%) at GD17.5 when compared with the CSA-NP_SCR_-treated mice (Supplemental Figure S2G).

Injection of the CD147 blocking antibody into the uterine lumen did not affect embryo implantation in mice (Supplemental Figure S3). On the other hand, placenta-specific suppression of CD147 induced fetal demise and extensive placental hemorrhage (Figure 1A), decreased alive litter size (Figure 1B) and body, placental and fetal weight (Figure 1C, D & E). The systolic blood pressure was elevated in the CSA-NP_CD147_-treated mice from GD9.5 to GD17.5 during pregnancy (Figure 1F). The urine protein/creatinine ratios (Figure 1G) and serum sFlt-1 level on GD17.5 (Figure 1H) were also higher in mice treated with the CSA-NP_CD147_ than those with the CSA-NP_SCR_. Moreover, renal damages including glomerular capillary endotheliosis and glomerular erythropenia were observed in the CSA-NP_CD147_ treated mice (Figure 1I). Placenta and fetal development were determined by a high-frequency ultrasound imaging system (Figure 1J). Compared to the CSA-NP_SCR_ group, the CSA-NP_CD147_ group exhibited deterioration of development in terms of placental diameter, placental thickness, biparietal diameter, crown-rump length and fetal heart rate (Figure 1J). These results indicated that the placenta-specific knockdown of CD147 induced PE-like phenotypes and fetal growth retardation in mice. Consistently, histopathological analysis revealed the size of the labyrinth zone and the number of trophoblast-specific protein alpha^+^ (TPBPA^+^) invasive trophoblast giant cells, which correspond to EVCT in humans^23^, was decreased in the CSA-NP_CD147_ group when compared to the control (Figure 1K).

**CD147 regulates trophoblast differentiation**

The reduced number of invasive trophoblast giant cells suggested defective trophoblast differentiation and/or invasion in the placenta-specific CD147 knockdown mice. In addition, single-cell analysis data of human cell landscape^20^ and at early maternal-fetal interface^21^ showed that CD147 was highly expressed in the CT of human chorionic villus and EVCT (Supplemental Figure S4A & B). Taken together, we hypothesized that CD147 regulated placental development in humans by modulating CT differentiation and EVCT functions. Physiological study of human trophoblast differentiation is almost impossible in the past due to the limited availability of primary CT in early pregnancy. This has recently become feasible with the development of the TSC^3^ and trophoblast organoids^9^.

Both human KRT7^+^GATA3^+^ TSC established from human primary CT (Figure 2A) and GATA3^+^AP2α^+^AP2γ^+^ trophoblast organoids from placenta villi tissue (Figure 2B) were capable of differentiating into EVCT and ST *in vitro* (Figure 2A & B)^3,8,9^. Consistent with the primary CT, CD147 was highly expressed in TSCs (Figure 2A) and trophoblast organoids (Figure 2B). The addition of CD147 blocking antibody inhibited EVCT differentiation from TSCs as shown by the reduced expression of EVCT markers HLAG, MMP2, ITGA5 and FN1 (Figure 2A). A similar suppressive effect of CD147 blocking antibody on EVCT differentiation was observed in the human trophoblast organoids (Figure 2B). On the other hand, CD147 blocking antibody had no effect on ST differentiation of the TSC and the trophoblast organoids (Figure 2A & B).

**CD147 regulates the spiral artery remodeling functions of EVCT**

The physiological relevance of CD147 in spiral artery remodeling of EVCT was studied *in vitro*. We confirmed the expression of CD147 on the surface of human primary EVCTs and JEG-3 cells (Supplemental Figure S1B). Suppression of CD147 functions in the primary EVCT by functional blocking antibody and JEG-3 cell by siRNA (Supplemental Figure S5A & B) reduced the invasiveness and MMP2 expression/activity of the cells (Figure 3A & B). Among the MMPs, MMP-2 and MMP-9 are the key gelatinases involved in invasion of the uterus by first trimester EVCTs ^24^. Their production and activities in trophoblasts peak in the first trimester ^25^ coinciding with the maximal invasive behavior of EVCT in vivo. A dysregulated production of MMP-2/MMP-9 could interfere the physiological placentation. However, while our results indicate the regulatory role of CD147 on MMP2 expression/activity, no such effect can be detected in this study for MMP9. In contrast, the treatment did not affect migration of the cells (Data not shown). On the other hand, treatment with CD147 stimulatory ligation antibody, which stimulates the biological activities of the molecule^14^, promoted EVCT invasion (Figure 3A).

It had been suggested that PE is associated with endothelial cell dysfunctions^26,27^. Reduced angiogenesis^28^ and increased vascular permeability^29^ are characteristic vascular responses in PE. HUVEC is a well-established model to study vascular homeostasis^30^. CD147 suppression reduced the integration of JEG-3 cells to the HUVEC endothelial cell network (Figure 3C). Treatment with recombinant CD147 increased the angiogenic ability (Figure 3D) and reduced the permeability (Figure 3E) of HUVEC. All these observations support the role of CD147 in regulating vascular remodeling activities of EVCT.

**Integrin β1 mediates CD147 induced EVCT invasion**

CD147 exists as protein complexes on human trophoblast^19^. Blue native gel electrophoresis was performed to further examine the membrane complexes of CD147 in human primary EVCT. The results showed that CD147 was associated with two membrane protein complexes with a size of 280kDa and 480kDa on EVCT (Figure 3F). CD147 suppression decreased the abundance of these complexes (Figure 3F). Nano-liquid chromatography-tandem mass spectrometry identified 93 proteins in the 280-kDa band and 70 proteins in the 480-kDa band (Figure 3F, Supplemental Table ST2). Reactome pathway analysis suggested that these complexes might mediate CD147 interaction, cell-cell communication, cell junction organization, signaling by receptor tyrosine kinase and Wnt-5A-dependent internalization (Supplemental Table ST2).

PE is characterized by hypertension and is associated with defective vascular remodeling. Intriguingly, our protein interaction analysis identified several hypertension/vascular remodeling-associated genes that were predicted to interact with CD147, such as sodium/potassium-transporting ATPase subunit alpha-1/ alpha-3/beta-3 ^31^, plasma membrane calcium-transporting ATPase 4 ^32^, talin-1 ^33^, alpha-enolase ^34^, and septin-2 ^35^. Our data also suggested that the biological activities of CD147 were mediated via Wnt/β-catenin signaling (Figure 3I-K), and Wnt signaling has been implicated in the regulation of blood pressure ^36^ and vascular remodeling ^37^.

Protein interaction network analysis suggested integrin β1 as an interacting partner of CD147 in the two membrane protein complexes. Integrin β1 was mainly expressed in EVCT (Supplemental Figure S4). The interaction of CD147 and integrin β1 in the JEG-3 cells and primary EVCTs was confirmed by colocalization analysis in immunostaining (Supplemental Figure S1C) and immunoprecipitation experiments (Figure 3G). The stimulatory effect of CD147 ligation antibody on EVCT invasion was abolished by integrin β1 downregulation using siRNA (Figure 3H, Supplemental Figure S5A), indicating that integrin β1 mediates the CD147 regulatory activities on EVCT invasion.

To study the role of Wnt/β-catenin signaling in mediating the CD147 activities in EVCT, the expression of the active β-catenin (de-phosphorylated form), total β-catenin, inactive GSK3β (phosphorylated form) and Axin2 were determined by Western blot analysis. CD147 suppression reduced the expression of active and total β-catenin while increased that of inactive GSK3β (Figure 3I). A similar suppressive effect was obtained by integrin β1 suppression (Figure 3J). Wnt activator (Wnt-3A) rescued the inhibitory effect of CD147 siRNA on JEG-3 invasion (Figure 3K). On the other hand, Wnt inhibitor (DKK-1) abolished the CD147 ligation antibody-induced invasion in JEG-3 cells and primary EVCTs.

**Reduced CD147 expression in chorionic villus and serum of early pregnant women who develop PE subsequently**

The demographics of the normotensive and the PE subjects involved are shown in Supplemental Table ST3. Compared to the normotensive group, women with PE presented with proteinuria and significantly higher blood pressure. The fetal birth weight and placenta weight were also significantly lower in the PE group. There were no differences in maternal age, parity and body mass index between the two groups. To study the role of CD147 in pregnancy, CVS and maternal serum samples were collected from women in early pregnancy (11–13+6 weeks of gestation) and stored until delivery. Compared with normal pregnancy, the expression of CD147 was reduced in the early pregnancy placental villi from women who presented with PE at delivery (Figure 4A). Reduced levels of soluble CD147 were also noted in the early pregnancy sera of the PE patients (Figure 4B). The reduction was significantly higher in the early-onset PE subgroups than in the normotensive group. Previous studies reported that the level of CD147 was down-regulated in the placenta^38^ but up-regulated in the serum^39^ of preeclamptic women. A key difference between the present and the previous studies was the time of sample collection; previous studies collected the samples at delivery^38^ or after 20 weeks of gestation^39^, whereas our serum samples were collected in early pregnancy (from 11 weeks to 13 weeks + 6 day of gestation) before the diagnosis of PE.

The reason for the reduced trophoblastic CD147 expression in PE remains unclear. It may be due to dysregulation of CD147 production. There is no reported gene mutation of CD147 in patients with PE to date. *BSG* (gene name of CD147) is located in chr19:571277-583494 (Cytogenetic region: 19p13.3). The relationships between genetic variants in *BSG* nearby regions and human diseases were accessed by the GWAS catalog. There were 3 reported genetic variants of the *BSG* gene that were risk factors for endometriosis (rs144824657-T, GCST004873), Alzheimer's disease (rs201850688-G, GCST005549) and insomnia (rs76360971-?, GCST90026658). No *BSG* genetic variants have yet been reported to be associated with PE. There was also no reported association of PE traits with chromosome 19 in the GWAS Catalog *(e.g. PMID 33239696)*. Epigenetic modifications affect the CD147 expression. For example, gene methylation modulates the CD147 expression and enhances the metabolism and metastasis of non-small-cell lung cancer cells during progression of the disease ^40,41^. However, no epigenetic regulation of CD147 has been reported to be associated with PE. The expression of CD147 was also regulated by various factors such as prostaglandin E2 (PGE2) ^42^, progesterone ^43^ and Cyp60 ^44^ in non-trophoblastic cells. Interestingly, the circulating levels of PGE2 ^45-47^ and progesterone ^48^ are reduced in preeclampsia. Further research is needed to clarify the links of these genetic, epigenetic and protein factors with the reduced CD147 expression in PE.

In contrast to CD147 expression in CVS samples, serum CD147 alone is not a good marker for early prediction of PE. Although many biomarkers have been proposed to predict and diagnose PE, no single biomarker is specific and sensitive enough to predict and/or diagnose PE ^22,49,50^, probably because PE is a multifactorial disease with varied clinical manifestations and poorly understood pathogenesis. Recent evidences also support the use of a combination of biomarkers to better diagnose PE ^51^. Thus, the identification of new markers, such as CD147, can contribute to a more specific diagnosis of PE as well as identification of new pathological mechanisms and therapeutic targets.

**Summary**

In summary, CD147 regulates the differentiation and spiral artery remodeling functions of EVCTs, and its deficiency contributes to the pathophysiology of PE (Figure 4C). The interaction of CD147/integrin-β1 complex with Wnt/β-catenin signaling mediates the CD147 activities on EVCT invasion. The reduced serum and villous CD147 levels in PE worth further investigation in a larger clinical trial.

**References**

1. Leung TY, Leung TN, Sahota DS, et al. Trends in maternal obesity and associated risks of adverse pregnancy outcomes in a population of Chinese women. *BJOG : an international journal of obstetrics and gynaecology*. Nov 2008;115(12):1529-37. doi:10.1111/j.1471-0528.2008.01931.x

2. Lee CL, Chiu PC, Hautala L, et al. Human chorionic gonadotropin and its free beta-subunit stimulate trophoblast invasion independent of LH/hCG receptor. Research Support, Non-U.S. Gov't. *Molecular and cellular endocrinology*. Aug 15 2013;375(1-2):43-52. doi:10.1016/j.mce.2013.05.009

3. Okae H, Toh H, Sato T, et al. Derivation of Human Trophoblast Stem Cells. *Cell Stem Cell*. Jan 4 2018;22(1):50-63 e6. doi:10.1016/j.stem.2017.11.004

4. Vijayan M, Lee CL, Wong VHH, et al. Decidual glycodelin-A polarizes human monocytes into a decidual macrophage-like phenotype through Siglec-7. *J Cell Sci*. Jul 23 2020;133(14)doi:10.1242/jcs.244400

5. Lee CL, Vijayan M, Wang X, et al. Glycodelin-A stimulates the conversion of human peripheral blood CD16-CD56bright NK cell to a decidual NK cell-like phenotype. *Hum Reprod*. Apr 1 2019;34(4):689-701. doi:10.1093/humrep/dey378

6. Lee CQ, Gardner L, Turco M, et al. What Is Trophoblast? A Combination of Criteria Define Human First-Trimester Trophoblast. *Stem Cell Reports*. Feb 9 2016;6(2):257-72. doi:10.1016/j.stemcr.2016.01.006

7. Apps R, Murphy SP, Fernando R, Gardner L, Ahad T, Moffett A. Human leucocyte antigen (HLA) expression of primary trophoblast cells and placental cell lines, determined using single antigen beads to characterize allotype specificities of anti-HLA antibodies. *Immunology*. May 2009;127(1):26-39. doi:IMM3019 [pii]10.1111/j.1365-2567.2008.03019.x

8. Gao X, Nowak-Imialek M, Chen X, et al. Establishment of porcine and human expanded potential stem cells. *Nat Cell Biol*. Jun 2019;21(6):687-699. doi:10.1038/s41556-019-0333-2

9. Turco MY, Gardner L, Kay RG, et al. Trophoblast organoids as a model for maternal-fetal interactions during human placentation. *Nature*. Dec 2018;564(7735):263-267. doi:10.1038/s41586-018-0753-3

10. Zhang B, Tan L, Yu Y, et al. Placenta-specific drug delivery by trophoblast-targeted nanoparticles in mice. *Theranostics*. 2018;8(10):2765-2781. doi:10.7150/thno.22904

11. Zhang B, Chen Z, Han J, Li M, Nayak NR, Fan X. Comprehensive Evaluation of the Effectiveness and Safety of Placenta-Targeted Drug Delivery Using Three Complementary Methods. *J Vis Exp*. Sep 10 2018;(139)doi:10.3791/58219

12. Igakura T, Kadomatsu K, Kaname T, et al. A null mutation in basigin, an immunoglobulin superfamily member, indicates its important roles in peri-implantation development and spermatogenesis. Research Support, Non-U.S. Gov't. *Developmental biology*. Feb 15 1998;194(2):152-65.

13. Kornek M, Popov Y, Libermann TA, Afdhal NH, Schuppan D. Human T cell microparticles circulate in blood of hepatitis patients and induce fibrolytic activation of hepatic stellate cells. *Hepatology*. Jan 2011;53(1):230-42. doi:10.1002/hep.23999

14. Koch C, Staffler G, Huttinger R, et al. T cell activation-associated epitopes of CD147 in regulation of the T cell response, and their definition by antibody affinity and antigen density. *Int Immunol*. May 1999;11(5):777-86.

15. Guo Y, Lee CL, So KH, et al. Soluble human leukocyte antigen-g5 activates extracellular signal-regulated protein kinase signaling and stimulates trophoblast invasion. *PloS one*. 2013;8(10):e76023. doi:10.1371/journal.pone.0076023

16. Lam KK, Chiu PC, Chung MK, et al. Glycodelin-A as a modulator of trophoblast invasion. *Hum Reprod*. Sep 2009;24(9):2093-103. doi:dep205 [pii]10.1093/humrep/dep205

17. Lee CL, Veerbeek JHW, Rana TK, van Rijn BB, Burton GJ, Yung HW. Role of Endoplasmic Reticulum Stress in Proinflammatory Cytokine-Mediated Inhibition of Trophoblast Invasion in Placenta-Related Complications of Pregnancy. *Am J Pathol*. Feb 2019;189(2):467-478. doi:10.1016/j.ajpath.2018.10.015

18. Xu B, Nakhla S, Makris A, Hennessy A. TNF-alpha inhibits trophoblast integration into endothelial cellular networks. *Placenta*. Mar 2011;32(3):241-6. doi:10.1016/j.placenta.2010.12.005

19. Lee CL, Lam MP, Lam KK, et al. Identification of CD147 (basigin) as a mediator of trophoblast functions. Research Support, Non-U.S. Gov't. *Human reproduction*. Nov 2013;28(11):2920-9. doi:10.1093/humrep/det355

20. Han X, Zhou Z, Fei L, et al. Construction of a human cell landscape at single-cell level. *Nature*. May 2020;581(7808):303-309. doi:10.1038/s41586-020-2157-4

21. Vento-Tormo R, Efremova M, Botting RA, et al. Single-cell reconstruction of the early maternal-fetal interface in humans. *Nature*. Nov 2018;563(7731):347-353. doi:10.1038/s41586-018-0698-6

22. Zhang J, Han L, Li W, et al. Early prediction of preeclampsia and small-for-gestational-age via multi-marker model in Chinese pregnancies: a prospective screening study. *BMC Pregnancy Childbirth*. Aug 19 2019;19(1):304. doi:10.1186/s12884-019-2455-8

23. Woods L, Perez-Garcia V, Hemberger M. Regulation of Placental Development and Its Impact on Fetal Growth-New Insights From Mouse Models. *Front Endocrinol (Lausanne)*. 2018;9:570. doi:10.3389/fendo.2018.00570

24. Gualdoni GS, Jacobo PV, Barril C, Ventureira MR, Cebral E. Early Abnormal Placentation and Evidence of Vascular Endothelial Growth Factor System Dysregulation at the Feto-Maternal Interface After Periconceptional Alcohol Consumption. *Front Physiol*. 2021;12:815760. doi:10.3389/fphys.2021.815760

25. Staun-Ram E, Goldman S, Gabarin D, Shalev E. Expression and importance of matrix metalloproteinase 2 and 9 (MMP-2 and -9) in human trophoblast invasion. *Reprod Biol Endocrinol*. Aug 4 2004;2:59. doi:10.1186/1477-7827-2-59

26. Granger JP, Alexander BT, Llinas MT, Bennett WA, Khalil RA. Pathophysiology of hypertension during preeclampsia linking placental ischemia with endothelial dysfunction. *Hypertension*. Sep 2001;38(3 Pt 2):718-22.

27. LaMarca BD, Gilbert J, Granger JP. Recent progress toward the understanding of the pathophysiology of hypertension during preeclampsia. *Hypertension*. Apr 2008;51(4):982-8. doi:10.1161/HYPERTENSIONAHA.107.108837

28. Ahmad S, Ahmed A. Elevated placental soluble vascular endothelial growth factor receptor-1 inhibits angiogenesis in preeclampsia. *Circ Res*. Oct 29 2004;95(9):884-91. doi:10.1161/01.RES.0000147365.86159.f5

29. Wang Y, Lewis DF, Alexander JS, Granger DN. Endothelial barrier function in preeclampsia. *Front Biosci*. Jan 1 2007;12:2412-24. doi:10.2741/2243

30. Onat D, Brillon D, Colombo PC, Schmidt AM. Human vascular endothelial cells: a model system for studying vascular inflammation in diabetes and atherosclerosis. *Curr Diab Rep*. Jun 2011;11(3):193-202. doi:10.1007/s11892-011-0182-2

31. Kaplan JH. The sodium pump and hypertension: a physiological role for the cardiac glycoside binding site of the Na,K-ATPase. *Proc Natl Acad Sci U S A*. Nov 1 2005;102(44):15723-4. doi:10.1073/pnas.0507965102

32. Little R, Cartwright EJ, Neyses L, Austin C. Plasma membrane calcium ATPases (PMCAs) as potential targets for the treatment of essential hypertension. *Pharmacol Ther*. Mar 2016;159:23-34. doi:10.1016/j.pharmthera.2016.01.013

33. Aoyama M, Kishimoto Y, Saita E, et al. High Plasma Levels of Soluble Talin-1 in Patients with Coronary Artery Disease. *Dis Markers*. 2020;2020:2479830. doi:10.1155/2020/2479830

34. Dai J, Zhou Q, Chen J, Rexius-Hall ML, Rehman J, Zhou G. Alpha-enolase regulates the malignant phenotype of pulmonary artery smooth muscle cells via the AMPK-Akt pathway. *Nat Commun*. Sep 21 2018;9(1):3850. doi:10.1038/s41467-018-06376-x

35. Neubauer K, Zieger B. Role of Septins in Endothelial Cells and Platelets. *Front Cell Dev Biol*. 2021;9:768409. doi:10.3389/fcell.2021.768409

36. Abou Ziki MD, Mani A. Wnt signaling, a novel pathway regulating blood pressure? State of the art review. *Atherosclerosis*. Jul 2017;262:171-178. doi:10.1016/j.atherosclerosis.2017.05.001

37. Corada M, Nyqvist D, Orsenigo F, et al. The Wnt/beta-catenin pathway modulates vascular remodeling and specification by upregulating Dll4/Notch signaling. *Dev Cell*. Jun 15 2010;18(6):938-49. doi:10.1016/j.devcel.2010.05.006

38. Wang YQ, Mi SF, Li J, Wang YL, Shang T. [Differential expression of extracellular matrix metalloproteinase inducer in normal placenta and preeclampsia placenta]. *Zhonghua fu chan ke za zhi*. Jul 2006;41(7):436-9.

39. Romao M, Weel IC, Lifshitz SJ, Peracoli MT. Elevated hyaluronan and extracellular matrix metalloproteinase inducer levels in women with preeclampsia. *Arch Gynecol Obstet*. Mar 2014;289(3):575-9. doi:10.1007/s00404-013-3021-7

40. Wang K, Huang W, Chen R, et al. Di-methylation of CD147-K234 Promotes the Progression of NSCLC by Enhancing Lactate Export. *Cell Metab*. Jan 5 2021;33(1):160-173 e6. doi:10.1016/j.cmet.2020.12.010

41. Liao CG, Liang XH, Ke Y, et al. Active demethylation upregulates CD147 expression promoting non-small cell lung cancer invasion and metastasis. *Oncogene*. Feb 7 2022;doi:10.1038/s41388-022-02213-0

42. Lee J, Banu SK, Subbarao T, Starzinski-Powitz A, Arosh JA. Selective inhibition of prostaglandin E2 receptors EP2 and EP4 inhibits invasion of human immortalized endometriotic epithelial and stromal cells through suppression of metalloproteinases. *Mol Cell Endocrinol*. Jan 30 2011;332(1-2):306-13. doi:10.1016/j.mce.2010.11.022

43. Xu Q, Ohara N, Liu J, et al. Progesterone receptor modulator CDB-2914 induces extracellular matrix metalloproteinase inducer in cultured human uterine leiomyoma cells. *Mol Hum Reprod*. Mar 2008;14(3):181-91. doi:10.1093/molehr/gan004

44. Pushkarsky T, Yurchenko V, Vanpouille C, et al. Cell surface expression of CD147/EMMPRIN is regulated by cyclophilin 60. *J Biol Chem*. Jul 29 2005;280(30):27866-71. doi:10.1074/jbc.M503770200

45. Pedersen EB, Christensen NJ, Christensen P, et al. Preeclampsia -- a state of prostaglandin deficiency? Urinary prostaglandin excretion, the renin-aldosterone system, and circulating catecholamines in preeclampsia. *Hypertension*. Jan-Feb 1983;5(1):105-11. doi:10.1161/01.hyp.5.1.105

46. Clark BA, Ludmir J, Epstein FH, et al. Urinary cyclic GMP, endothelin, and prostaglandin E2 in normal pregnancy and preeclampsia. *Am J Perinatol*. Oct 1997;14(9):559-62. doi:10.1055/s-2007-994334

47. Vural P, Akgul C, Canbaz M. Urinary PGE2 and PGF2alpha levels and renal functions in preeclampsia. *Gynecol Obstet Invest*. 1998;45(4):237-41. doi:10.1159/000009975

48. Wan J, Hu Z, Zeng K, et al. The reduction in circulating levels of estrogen and progesterone in women with preeclampsia. *Pregnancy Hypertens*. Jan 2018;11:18-25. doi:10.1016/j.preghy.2017.12.003

49. Agrawal S, Shinar S, Cerdeira AS, Redman C, Vatish M. Predictive Performance of PlGF (Placental Growth Factor) for Screening Preeclampsia in Asymptomatic Women: A Systematic Review and Meta-Analysis. *Hypertension*. Nov 2019;74(5):1124-1135. doi:10.1161/HYPERTENSIONAHA.119.13360

50. Grill S, Rusterholz C, Zanetti-Dallenbach R, et al. Potential markers of preeclampsia--a review. *Reprod Biol Endocrinol*. Jul 14 2009;7:70. doi:10.1186/1477-7827-7-70

51. Stepan H, Hund M, Andraczek T. Combining Biomarkers to Predict Pregnancy Complications and Redefine Preeclampsia: The Angiogenic-Placental Syndrome. *Hypertension*. Apr 2020;75(4):918-926. doi:10.1161/HYPERTENSIONAHA.119.13763

**List of abbreviations**

BNG Blue native gel

BP Blood pressure

CSA-NP_CD147_ plCSA-BP-modified CD147 Morpholino CD147-Mor nanoparticles

CSA-NP_CD147-GFP_ plCSA-BP-modified Green-fluorescent modification of CD147 Morpholino nanoparticles

CSA-NP_SCR_ plCSA-BP-modified CD147-mispair scramble morpholino nanoparticles

CT Cytotrophoblast

CVS Chorionic villus sampling

DAPI 4′,6-diamidino-2-phenylindole

DMEM Dulbecco's Modified Eagle Medium

ELISA Enzyme-linked immunosorbent assay

EVCT Extravillous trophoblast

FGR Fetal growth restriction

GD Gestational day

HLA-G Human leukocyte antigen G

HUVEC Human umbilical vein endothelial cell

PE Preeclampsia

plCSA-BP Placental chondroitin sulfate A binding peptide

PlGF Placental growth factor

SD Standard deviation

sFlt-1 Soluble fms-like tyrosine kinase-1

ST Syncytiotrophoblast

TPBPA Trophoblast-specific protein alpha

TSC Trophoblast stem cells

qPCR quantitative real-time PCR

**Supplemental Figure S1.** Expression of CD147 in (A) Human (term) placenta, mouse (GD16.5) placenta, (B) Primary EVCT and JEG-3 cells. (C) Co-expression of CD147 and integrin β1 in JEG-3 and EVCT (N=3).

**
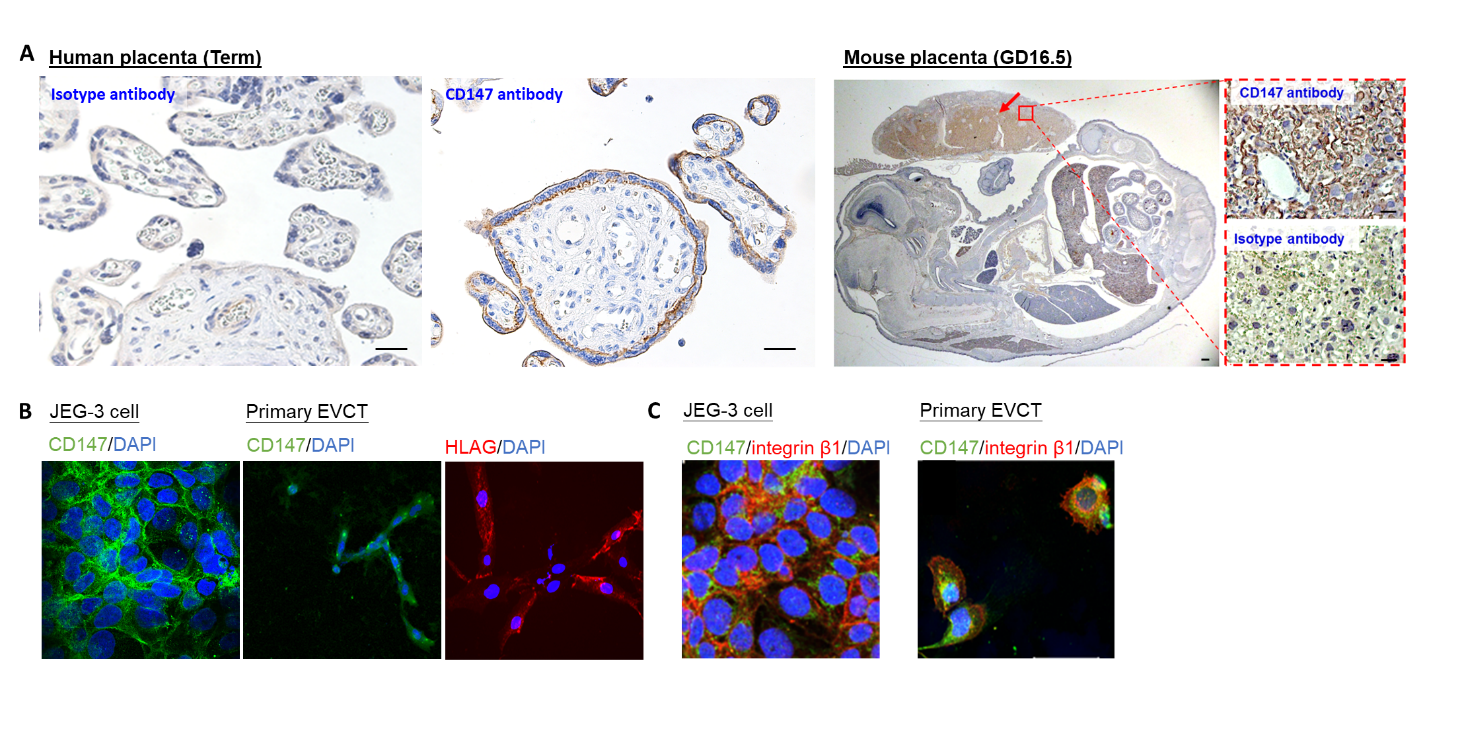
**

**Supplemental Figure S2.** Synthesis and characterization of placenta-specific nanoparticles encapsulated CD147 morpholino. (A) Schematic diagram of the preparation of CD147 morpholino nanoparticles using the single-step sonication method and conjugating the nanoparticles with CSA peptides. (B) Size distributions of CSA-NP_SCR_ and CSA-NP_CD147_ were determined by dynamic light scattering (N=3). (C) Mouse migratory trophoblast cells (MTB) cells treated with plCSA-Mor-NPs, and mouse 3T3-L1 cell (fibroblasts) treated with CSA-NP_GFP_ (negative control). (D) The synthesized CSA-NP_SCR_ and CSA-NP_CD147_ nanoparticles have no cytotoxic effect on the MTB cells by CCK8 assay (N=6). (E) Protein expression level of CD147 in mouse placenta. (F) Schematic of pregnant mice injected with CSA-NP_SCR_ and CSA-NP_CD147_. Uptake of placenta-specific CSA-NP_CD147-GFP_ nanoparticles in mice placentas was examined by fluorescent microscope (N=3). Scale bar = 100 & 200μm. CK8: cytokeratin 8 (mouse trophoblast marker), DE: decidua, JZ: junctional zone, LZ: labyrinth zone (G) The CD147 mRNA and protein expression in mouse placentas on GD14.5 were determined after treatment of CSA-NP_SCR_ or CSA-NP_CD147_. β-actin as an internal control (N=3). The CD147 protein expression in the junctional and labyrinth zone of mouse placentas on GD17.5 were suppressed by CSA-NP_CD147_ compared to CSA-NP_SCR_ group. Scale bar = 350μm. All the data are expressed as mean ± SD. *** p<0.001.


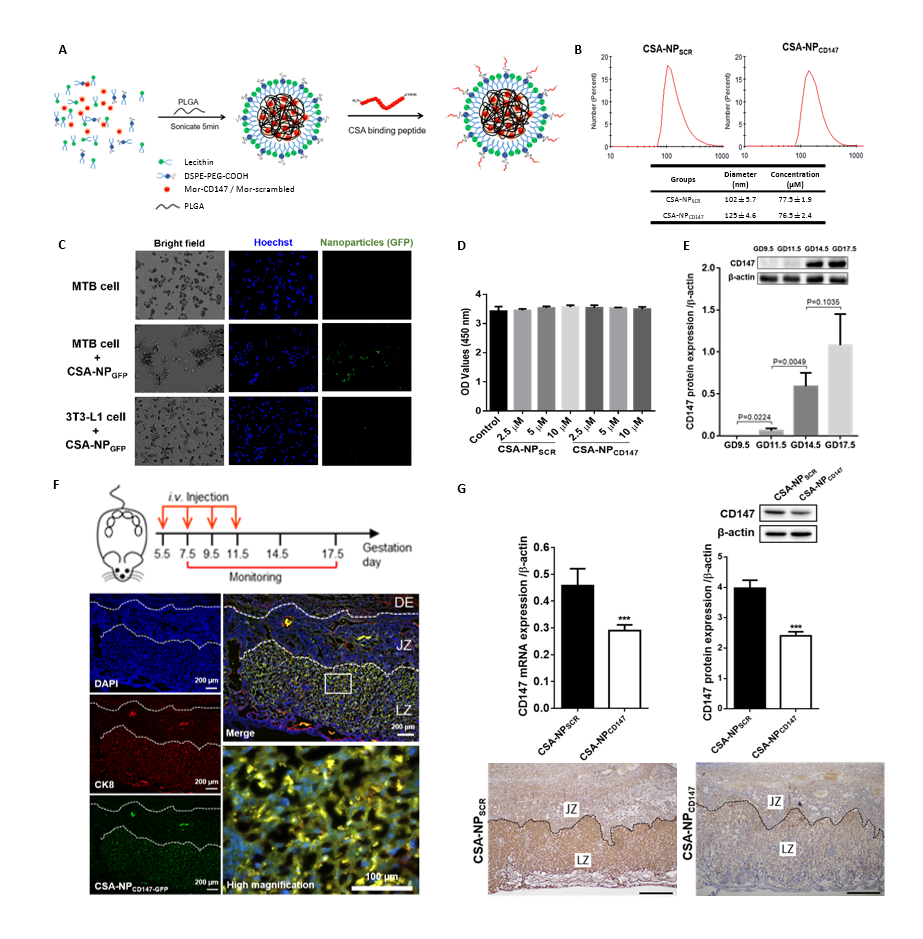


**Supplemental Figure S3.** Blocking of CD147 on mouse blastocyst and endometrium has no effect on implantation. Mouse embryos transfect to pseudo-pregnant female mice with control and CD147 blocking antibodies (N=4).


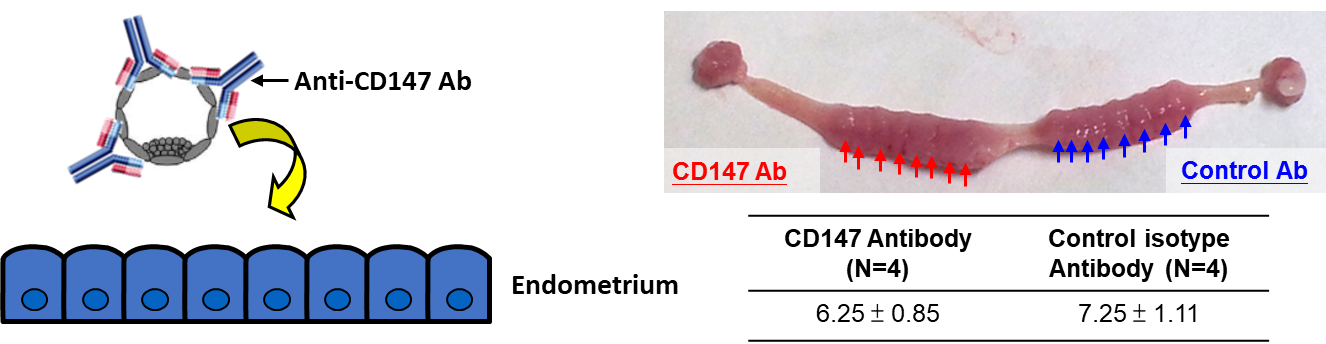


**Supplemental Figure S4.** Protein expression pattern of CD147 and integrin β1 in human tissues. (A) Expression of CD147 and integrin β1 in human tissues. (B) Expression of CD147 and integrin β1 in the feto-maternal interface.


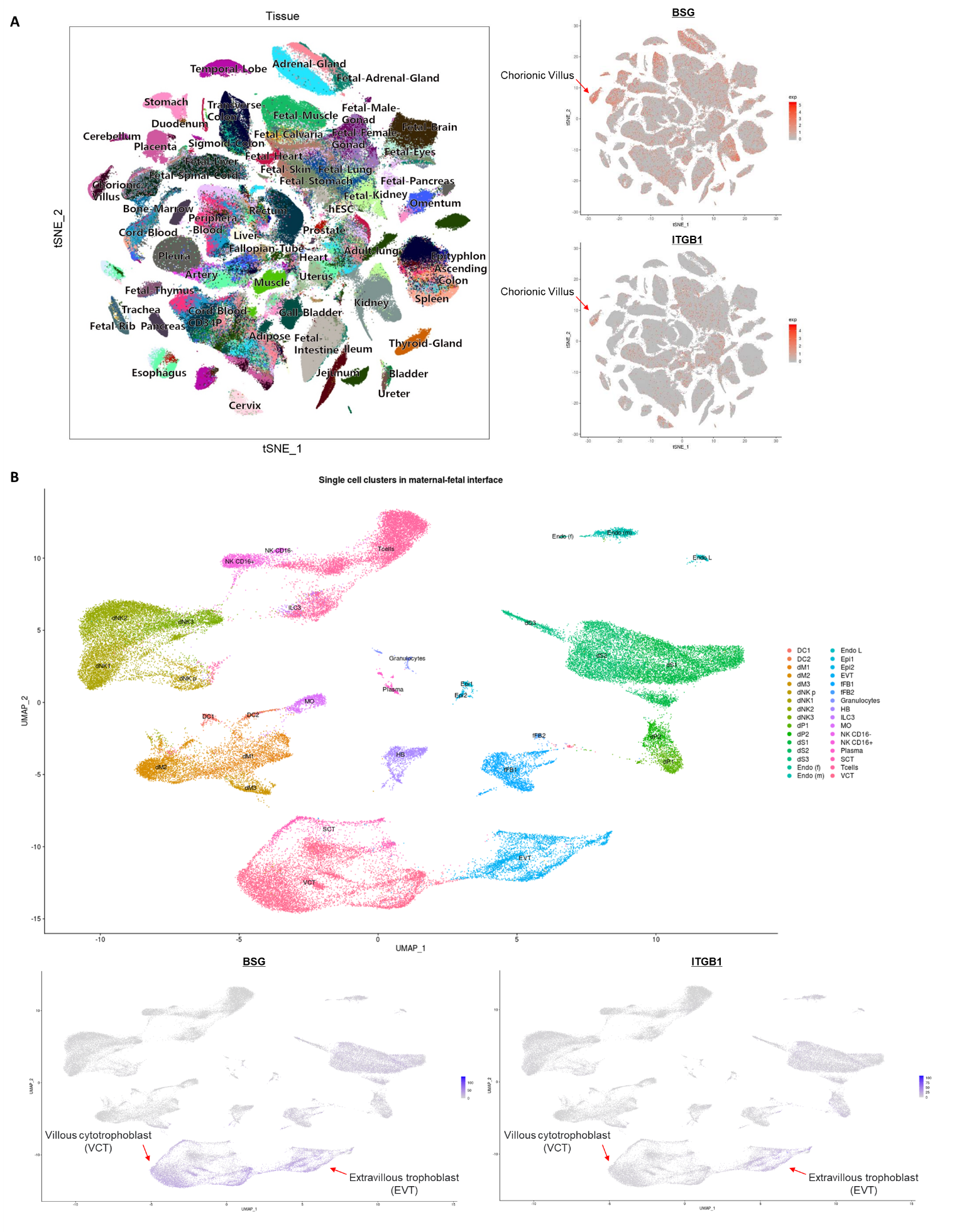


**Supplemental Figure S5. Suppression of CD147 and integrin β1 by siRNA in JEG-3 cells. (A)** Protein expression of CD147 (N=5) and integrin β1 (N=3) after siRNA treatment by western blot. Transfection efficiency was shown as the percentage of the negative siRNA control. **(B)** The viability of JEG-3 cells was not affected by control and CD147 siRNA suppression at 24 and 48h (N=5). All the data are expressed as mean ± SD.

**
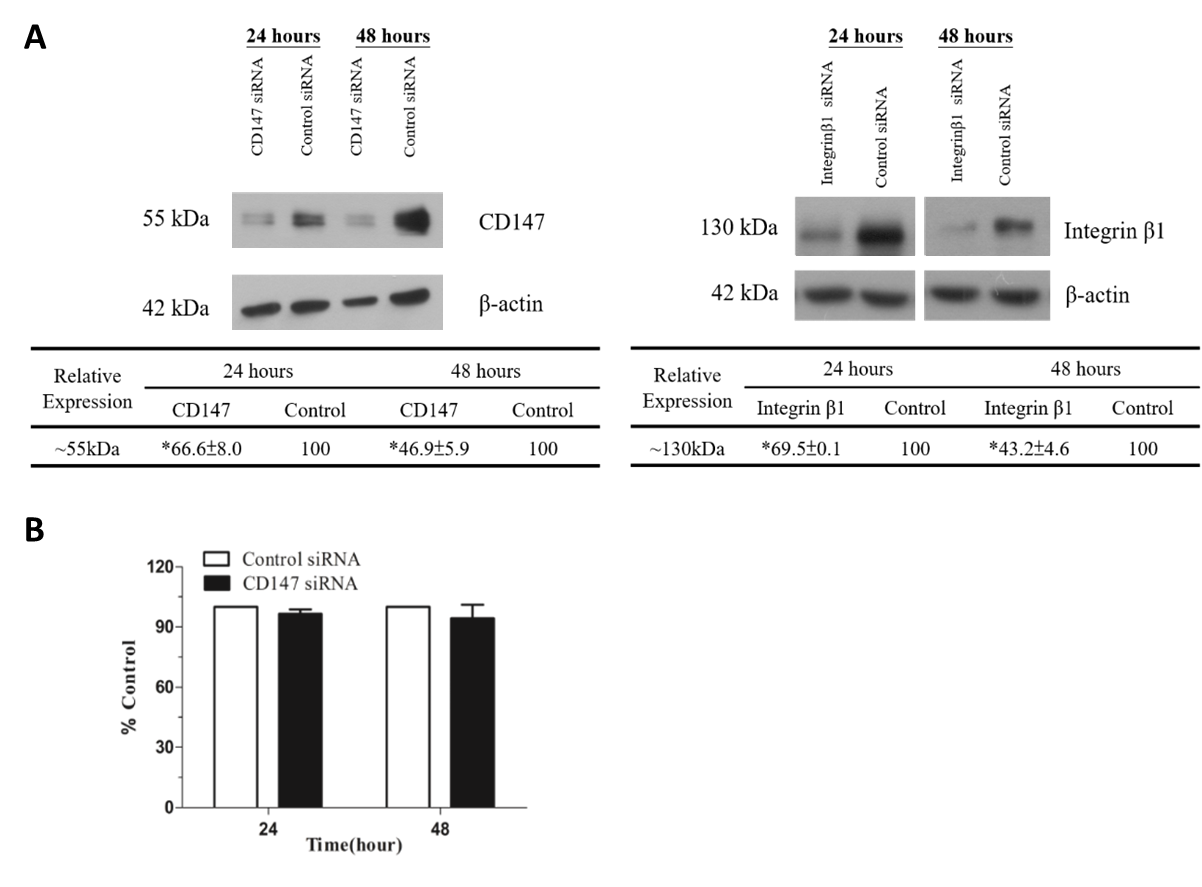
**

**Supplemental Table ST1.** List of antibodies used in this study.

| **Antibody** | **Conjugate** | **Manufacturer** | **Cat. No.** | **Clone** | **Dilution** |
| --- | --- | --- | --- | --- | --- |
| KRT7 | AF488 | Abcam | ab208273 | EPR17078 | 1:100 |
| KRT7 | Alexa Fluor® 555 | Abcam | ab209601 | EPR17078 | 1:100 |
| HLA-G | - | Abcam | ab7758 | MEM-G/9 | 1:100 (Immunofluorescence) |
|  |  |  |  |  | 1:100 (Flow cytometry) |
| CD49f | PE | Miltenyi Biotec | 130-097-246 | GoH3 | 1:100 |
| MCT4 | - | Merck Millipore | AB3314P | - | 1:100 |
| Laminin | - | Sigma | L9393 | - | 1:100 |
| TPBPA | - | Abcam | ab104401 | - | 1:100 |
| GATA3 | Alexa Fluor® 488 | Abcam | ab208895 | EPR16651 | 1:100 (Immunofluorescence) |
|  | - | Abcam | ab199428 | EPR16651 | 1:100 (Immunohistochemistry) |
| GCM1 | - | Abcam | ab187860 | - | 1:100 |
| hCG | - | Abcam | ab11388 | INN-hCG-2 | 1:100 |
| AP2α | - | Abcam | ab108311 | EPR2688(2) | 1:100 |
| AP2γ | - | Abcam | ab110635 | 3B5 | 1:50 |
| CD147 | - | Abcam | ab64616 | - | 1:100 (Immunofluorescence) |
| CD147 | - | Abcam | ab666 | MEM-M6/1 | 1:100 (Immunohistochemistry) |
|  |  |  |  |  | 1:1000 (Western blot) |
|  |  |  |  |  | 1µg/mL (Flow cytometry) |
| CD147 |  | Abcam | ab11572 | - | 1:100 (Immunoprecipitation) |
| CD147 | - | Abcam | ab49493 | AB1843 | 5µg/mL (Functional blocking Ab) |
| CD147 |  | Abcam | ab232967 | - | 5µg/mL (Functional blocking Ab) |
|  |  |  |  | - | 1µg/mL (Western blot) |
| CD147 | - | Abcam | ab119114 | MEM-M6/6 | 5µg/mL (Stimulatory ligation Ab) |
|  |  |  |  |  | 1:20 (Electronic microscopy) |
| Integrin β1 | - | Abcam | ab30394 | 12G10 | 1:1000 (Western blot) |
|  |  |  |  |  | 1:100 (Immunofluorescence) |
| Active β-catenin | - | Millipore | 05-665 | 8E7 | 1:1000 |
| Total β-catenin | - | BD Biosciences | 610153 | 14/β-catenin | 1:5000 |
| GSK-3β | - | Abcam | ab75814 | EPR2286Y | 1:5000 |
| Axin2 | - | Abcam | ab32197 | - | 1 µg/mL |
| β-Actin | - | Sigma-Aldrich | A3854 | AC-15 | 1:10000 |
| α-Tubulin | - | Abcam | ab7291 | DM1A | 1:10000 |
| mouse IgG isotype control | - | Merck Millipore | 12-371 | - | Assay dependent |
| mouse IgG isotype control | - | Abcam | ab18449 | MOPC-173 | Assay dependent |
| rabbit IgG isotype control | - | Abcam | ab172730 | EPR25A | Assay dependent |
| mouse IgG (H+L) | AF488 | Thermo Fisher | A21202 | - | 1:1000 |
| mouse IgG (H+L) | PE | Thermo Fisher | PA1-84395 | - | 1:2000 |
| mouse IgG (H+L) | AF647 | Thermo Fisher | A31571 | - | 1:1000 |
| DAPI | - | Thermo Fisher | D1306 | - | 1µM |

**Supplemental Table ST2.** Identification of the 280 and 480kDa CD147 protein complexes in EVCTs by LC-MS/MS. The acquired data were analysed using the ProteinPilot software (AB Sciex). The spectra were matched against the Uniprot Homo Sapiens transmembrane proteome subset database. Peptides were identified based on the 99% confidence (Unused Score >2) of sequence matching.

**280kDa Complex**

| **N** | **Unused** | **Total** | **%Cov** | **Name / Species / Gene Name** | **Accession** |
| --- | --- | --- | --- | --- | --- |
| 1 | 132.38 | 132.38 | 83.74 | Alpha-actinin-1 OS=Homo sapiens GN=ACTN1 PE=1 SV=2 | sp\|P12814\|ACTN1_HUMAN |
| 2 | 124.26 | 124.26 | 67.9 | Heat shock protein HSP 90-alpha OS=Homo sapiens GN=HSP90AA1 PE=1 SV=5 | sp\|P07900\|HS90A_HUMAN |
| 3 | 66.58 | 66.58 | 62.54 | Heat shock cognate 71 kDa protein OS=Homo sapiens GN=HSPA8 PE=1 SV=1 | sp\|P11142\|HSP7C_HUMAN |
| 4 | 62.27 | 62.27 | 38.51 | Sodium/potassium-transporting ATPase subunit alpha-1 OS=Homo sapiens GN=ATP1A1 PE=1 SV=1 | sp\|P05023\|AT1A1_HUMAN |
| 5 | 50.58 | 50.58 | 32.15 | Plasma membrane calcium-transporting ATPase 4 OS=Homo sapiens GN=ATP2B4 PE=1 SV=2 | sp\|P23634\|AT2B4_HUMAN |
| 6 | 49.87 | 49.96 | 22.9 | Talin-1 OS=Homo sapiens GN=TLN1 PE=1 SV=3 | sp\|Q9Y490\|TLN1_HUMAN |
| 7 | 40.7 | 40.7 | 33.65 | 4F2 cell-surface antigen heavy chain OS=Homo sapiens GN=SLC3A2 PE=1 SV=3 | sp\|P08195\|4F2_HUMAN |
| 8 | 27.18 | 27.18 | 28.95 | Transferrin receptor protein 1 OS=Homo sapiens GN=TFRC PE=1 SV=2 | sp\|P02786\|TFR1_HUMAN |
| 9 | 25.18 | 25.18 | 29.47 | Catenin alpha-1 OS=Homo sapiens GN=CTNNA1 PE=1 SV=1 | sp\|P35221\|CTNA1_HUMAN |
| 10 | 23.42 | 23.42 | 20.7 | AP-2 complex subunit beta OS=Homo sapiens GN=AP2B1 PE=1 SV=1 | sp\|P63010\|AP2B1_HUMAN |
| 11 | 22.02 | 22.02 | 43.78 | Alpha-enolase OS=Homo sapiens GN=ENO1 PE=1 SV=2 | sp\|P06733\|ENOA_HUMAN |
| 12 | 19.78 | 19.78 | 21.51 | Catenin beta-1 OS=Homo sapiens GN=CTNNB1 PE=1 SV=1 | sp\|P35222\|CTNB1_HUMAN |
| 13 | 19.47 | 19.5 | 45.94 | Voltage-dependent anion-selective channel protein 1 OS=Homo sapiens GN=VDAC1 PE=1 SV=2 | sp\|P21796\|VDAC1_HUMAN |
| 14 | 19.26 | 19.35 | 20.43 | Integrin beta-1 OS=Homo sapiens GN=ITGB1 PE=1 SV=2 | sp\|P05556\|ITB1_HUMAN |
| 15 | 18.25 | 18.27 | 44.42 | Aspartate aminotransferase, mitochondrial OS=Homo sapiens GN=GOT2 PE=1 SV=3 | sp\|P00505\|AATM_HUMAN |
| 16 | 18.21 | 18.27 | 18.22 | AP-2 complex subunit alpha-1 OS=Homo sapiens GN=AP2A1 PE=1 SV=3 | sp\|O95782\|AP2A1_HUMAN |
| 17 | 18.02 | 18.02 | 23.61 | Minor histocompatibility antigen H13 OS=Homo sapiens GN=HM13 PE=1 SV=1 | sp\|Q8TCT9\|HM13_HUMAN |
| 18 | 16.99 | 17.08 | 9.495 | Integrin beta-4 OS=Homo sapiens GN=ITGB4 PE=1 SV=5 | sp\|P16144\|ITB4_HUMAN |
| 19 | 16.49 | 16.51 | 37.67 | Septin-2 OS=Homo sapiens GN=SEPT2 PE=1 SV=1 | sp\|Q15019\|SEPT2_HUMAN |
| 20 | 16.31 | 16.33 | 21.16 | Vinculin OS=Homo sapiens GN=VCL PE=1 SV=4 | sp\|P18206\|VINC_HUMAN |
| 21 | 14.11 | 14.11 | 22.08 | Basigin OS=Homo sapiens GN=BSG PE=1 SV=2 | sp\|P35613\|BASI_HUMAN |
| 22 | 14 | 14 | 14.61 | ATP-binding cassette sub-family B member 6, mitochondrial OS=Homo sapiens GN=ABCB6 PE=1 SV=1 | sp\|Q9NP58\|ABCB6_HUMAN |
| 23 | 13.52 | 13.54 | 16.46 | Solute carrier family 2, facilitated glucose transporter member 1 OS=Homo sapiens GN=SLC2A1 PE=1 SV=2 | sp\|P11166\|GTR1_HUMAN |
| 24 | 12.59 | 12.61 | 41.94 | Sodium/potassium-transporting ATPase subunit beta-3 OS=Homo sapiens GN=ATP1B3 PE=1 SV=1 | sp\|P54709\|AT1B3_HUMAN |
| 25 | 12.01 | 12.1 | 5.214 | Nesprin-2 OS=Homo sapiens GN=SYNE2 PE=1 SV=3 | sp\|Q8WXH0\|SYNE2_HUMAN |
| 26 | 10.98 | 10.99 | 22.24 | ATP synthase subunit alpha, mitochondrial OS=Homo sapiens GN=ATP5A1 PE=1 SV=1 | sp\|P25705\|ATPA_HUMAN |
| 27 | 10.74 | 10.78 | 12.67 | Ras GTPase-activating-like protein IQGAP1 OS=Homo sapiens GN=IQGAP1 PE=1 SV=1 | sp\|P46940\|IQGA1_HUMAN |
| 28 | 10.51 | 10.55 | 28.31 | Transmembrane emp24 domain-containing protein 10 OS=Homo sapiens GN=TMED10 PE=1 SV=2 | sp\|P49755\|TMEDA_HUMAN |
| 29 | 10.37 | 10.4 | 51.32 | Protein deglycase DJ-1 OS=Homo sapiens GN=PARK7 PE=1 SV=2 | sp\|Q99497\|PARK7_HUMAN |
| 30 | 10.31 | 10.4 | 3.147 | Prolow-density lipoprotein receptor-related protein 1 OS=Homo sapiens GN=LRP1 PE=1 SV=2 | sp\|Q07954\|LRP1_HUMAN |
| 31 | 10.28 | 10.38 | 12.58 | Protein diaphanous homolog 1 OS=Homo sapiens GN=DIAPH1 PE=1 SV=2 | sp\|O60610\|DIAP1_HUMAN |
| 32 | 10.22 | 10.25 | 15 | Monocarboxylate transporter 1 OS=Homo sapiens GN=SLC16A1 PE=1 SV=3 | sp\|P53985\|MOT1_HUMAN |
| 33 | 8.71 | 8.7 | 18.9 | Anoctamin-6 OS=Homo sapiens GN=ANO6 PE=1 SV=2 | sp\|Q4KMQ2\|ANO6_HUMAN |
| 34 | 8.62 | 8.63 | 3.528 | Lipopolysaccharide-responsive and beige-like anchor protein OS=Homo sapiens GN=LRBA PE=1 SV=4 | sp\|P50851\|LRBA_HUMAN |
| 35 | 8.3 | 8.33 | 23.37 | Adenylyl cyclase-associated protein 1 OS=Homo sapiens GN=CAP1 PE=1 SV=5 | sp\|Q01518\|CAP1_HUMAN |
| 36 | 8.14 | 8.15 | 25.75 | AP-2 complex subunit mu OS=Homo sapiens GN=AP2M1 PE=1 SV=2 | sp\|Q96CW1\|AP2M1_HUMAN |
| 37 | 7.1 | 7.15 | 18.32 | Keratin, type II cytoskeletal 1 OS=Homo sapiens GN=KRT1 PE=1 SV=6 | sp\|P04264\|K2C1_HUMAN |
| 38 | 6.55 | 6.57 | 28.14 | 40S ribosomal protein SA OS=Homo sapiens GN=RPSA PE=3 SV=1 |  |
| 39 | 6.49 | 6.57 | 9.646 | Integrin alpha-6 OS=Homo sapiens GN=ITGA6 PE=1 SV=5 | sp\|P23229\|ITA6_HUMAN |
| 40 | 6.04 | 6.04 | 12.82 | Large neutral amino acids transporter small subunit 1 OS=Homo sapiens GN=SLC7A5 PE=1 SV=2 | sp\|Q01650\|LAT1_HUMAN |
| 41 | 5.96 | 6.01 | 6.198 | Lysine--tRNA ligase OS=Homo sapiens GN=KARS PE=1 SV=3 | sp\|Q15046\|SYK_HUMAN |
| 42 | 5.82 | 5.85 | 8.387 | Monocarboxylate transporter 4 OS=Homo sapiens GN=SLC16A3 PE=1 SV=1 | sp\|O15427\|MOT4_HUMAN |
| 43 | 5.51 | 5.56 | 4.959 | Epidermal growth factor receptor OS=Homo sapiens GN=EGFR PE=1 SV=2 | sp\|P00533\|EGFR_HUMAN |
| 44 | 5.37 | 5.4 | 9.545 | Protein disulfide-isomerase A6 OS=Homo sapiens GN=PDIA6 PE=1 SV=1 | sp\|Q15084\|PDIA6_HUMAN |
| 45 | 5.33 | 5.45 | 10.31 | Equilibrative nucleoside transporter 1 OS=Homo sapiens GN=SLC29A1 PE=1 SV=3 | sp\|Q99808\|S29A1_HUMAN |
| 46 | 5.26 | 5.33 | 16.89 | Ezrin OS=Homo sapiens GN=EZR PE=1 SV=4 | sp\|P15311\|EZRI_HUMAN |
| 47 | 4.91 | 4.95 | 16.31 | Lysosome-associated membrane glycoprotein 1 OS=Homo sapiens GN=LAMP1 PE=1 SV=3 | sp\|P11279\|LAMP1_HUMAN |
| 48 | 4.81 | 4.86 | 11.19 | Trophoblast glycoprotein OS=Homo sapiens GN=TPBG PE=1 SV=1 | sp\|Q13641\|TPBG_HUMAN |
| 49 | 4.38 | 4.47 | 3.919 | Agrin OS=Homo sapiens GN=AGRN PE=1 SV=5 | sp\|O00468\|AGRIN_HUMAN |
| 50 | 4.29 | 4.32 | 5.435 | Extended synaptotagmin-1 OS=Homo sapiens GN=ESYT1 PE=1 SV=1 | sp\|Q9BSJ8\|ESYT1_HUMAN |
| 51 | 4.23 | 4.25 | 9.057 | Neutral amino acid transporter B(0) OS=Homo sapiens GN=SLC1A5 PE=1 SV=2 | sp\|Q15758\|AAAT_HUMAN |
| 52 | 4.1 | 4.11 | 16.9 | AP-2 complex subunit sigma OS=Homo sapiens GN=AP2S1 PE=1 SV=2 | sp\|P53680\|AP2S1_HUMAN |
| 53 | 4.03 | 4.04 | 8.768 | E-cadherin OS=Homo sapiens PE=2 SV=1 |  |
| 54 | 4 | 4.01 | 5.042 | Calpain-1 catalytic subunit OS=Homo sapiens GN=CAPN1 PE=1 SV=1 | sp\|P07384\|CAN1_HUMAN |
| 55 | 4 | 4 | 2.857 | Plexin-D1 OS=Homo sapiens GN=PLXND1 PE=1 SV=3 | sp\|Q9Y4D7\|PLXD1_HUMAN |
| 56 | 4 | 4 | 12.73 | Claudin-6 OS=Homo sapiens GN=CLDN6 PE=1 SV=2 | sp\|P56747\|CLD6_HUMAN |
| 57 | 3.9 | 3.97 | 19.06 | Junctional adhesion molecule A OS=Homo sapiens GN=F11R PE=1 SV=1 | sp\|Q9Y624\|JAM1_HUMAN |
| 58 | 3.26 | 3.31 | 8.864 | Cell cycle control protein 50A OS=Homo sapiens GN=TMEM30A PE=1 SV=1 | sp\|Q9NV96\|CC50A_HUMAN |
| 59 | 2.96 | 3 | 4.376 | Solute carrier family 12 member 9 OS=Homo sapiens GN=SLC12A9 PE=1 SV=1 | sp\|Q9BXP2\|S12A9_HUMAN |
| 60 | 2.85 | 2.89 | 9.309 | Nck-associated protein 1 OS=Homo sapiens GN=NCKAP1 PE=1 SV=1 | sp\|Q9Y2A7\|NCKP1_HUMAN |
| 61 | 2.8 | 2.84 | 6.947 | Long-chain fatty acid transport protein 6 OS=Homo sapiens GN=SLC27A6 PE=2 SV=1 | sp\|Q9Y2P4\|S27A6_HUMAN |
| 62 | 2.64 | 2.67 | 4.804 | Dystroglycan OS=Homo sapiens GN=DAG1 PE=1 SV=2 | sp\|Q14118\|DAG1_HUMAN |
| 63 | 2.38 | 2.39 | 9.548 | Neuroplastin OS=Homo sapiens GN=NPTN PE=1 SV=2 | sp\|Q9Y639\|NPTN_HUMAN |
| 64 | 2.35 | 2.37 | 9.313 | Unconventional myosin-Ic OS=Homo sapiens GN=MYO1C PE=1 SV=4 | sp\|O00159\|MYO1C_HUMAN |
| 65 | 2.29 | 2.31 | 15.28 | Ras-related protein Rab-11A OS=Homo sapiens GN=RAB11A PE=1 SV=3 | sp\|P62491\|RB11A_HUMAN |
| 66 | 2.26 | 2.28 | 8.453 | Plastin-2 OS=Homo sapiens GN=LCP1 PE=1 SV=6 | sp\|P13796\|PLSL_HUMAN |
| 67 | 2.25 | 2.27 | 3.046 | Protein arginine N-methyltransferase 8 OS=Homo sapiens GN=PRMT8 PE=1 SV=2 | sp\|Q9NR22\|ANM8_HUMAN |
| 68 | 2.11 | 2.23 | 9.928 | Constitutive coactivator of PPAR-gamma-like protein 1 OS=Homo sapiens GN=FAM120A PE=1 SV=2 | sp\|Q9NZB2\|F120A_HUMAN |
| 69 | 2.06 | 2.07 | 5.367 | Guanine nucleotide-binding protein G(i) subunit alpha-1 OS=Homo sapiens GN=GNAI1 PE=1 SV=2 | sp\|P63096\|GNAI1_HUMAN |
| 70 | 2.02 | 2.02 | 0.597 | Dystrophin OS=Homo sapiens GN=DMD PE=1 SV=3 | sp\|P11532\|DMD_HUMAN |
| 71 | 2.02 | 2.02 | 8.571 | Protein XRP2 OS=Homo sapiens GN=RP2 PE=1 SV=4 | sp\|O75695\|XRP2_HUMAN |
| 72 | 2.01 | 2.01 | 7.187 | Olfactory receptor 51E2 OS=Homo sapiens GN=OR51E2 PE=2 SV=1 | sp\|Q9H255\|O51E2_HUMAN |
| 73 | 2 | 26.68 | 19.05 | Sodium/potassium-transporting ATPase subunit alpha-3 OS=Homo sapiens GN=ATP1A3 PE=1 SV=3 | sp\|P13637\|AT1A3_HUMAN |
| 74 | 2 | 25.69 | 18.28 | Plasma membrane calcium-transporting ATPase 1 OS=Homo sapiens GN=ATP2B1 PE=1 SV=3 | sp\|P20020\|AT2B1_HUMAN |
| 75 | 2 | 2.01 | 2.822 | Sodium/hydrogen exchanger 1 OS=Homo sapiens GN=SLC9A1 PE=1 SV=2 | sp\|P19634\|SL9A1_HUMAN |
| 76 | 2 | 2 | 2.181 | Tubulin-specific chaperone D OS=Homo sapiens GN=TBCD PE=1 SV=2 | sp\|Q9BTW9\|TBCD_HUMAN |
| 77 | 2 | 2 | 1.877 | Misshapen-like kinase 1 OS=Homo sapiens GN=MINK1 PE=1 SV=2 | sp\|Q8N4C8\|MINK1_HUMAN |
| 78 | 2 | 2 | 13.12 | Prostasin OS=Homo sapiens GN=PRSS8 PE=1 SV=1 | sp\|Q16651\|PRSS8_HUMAN |
| 79 | 2 | 2 | 16.75 | Ras-related protein Rab-13 OS=Homo sapiens GN=RAB13 PE=1 SV=1 | sp\|P51153\|RAB13_HUMAN |
| 80 | 2 | 2 | 8.011 | Putative HLA class I histocompatibility antigen, alpha chain H OS=Homo sapiens GN=HLA-H PE=5 SV=3 | sp\|P01893\|HLAH_HUMAN |
| 80 | 0 | 2 | 6.557 | HLA class I histocompatibility antigen, Cw-16 alpha chain OS=Homo sapiens GN=HLA-C PE=1 SV=1 | sp\|Q29960\|1C16_HUMAN |
| 81 | 2 | 2 | 8.846 | Carbonic anhydrase 2 OS=Homo sapiens GN=CA2 PE=1 SV=2 | sp\|P00918\|CAH2_HUMAN |
| 82 | 2 | 2 | 3.523 | Leucine zipper putative tumor suppressor 1 OS=Homo sapiens GN=LZTS1 PE=1 SV=3 | sp\|Q9Y250\|LZTS1_HUMAN |
| 83 | 2 | 2 | 7.309 | Cytoskeleton-associated protein 4 OS=Homo sapiens GN=CKAP4 PE=1 SV=2 | sp\|Q07065\|CKAP4_HUMAN |
| 84 | 2 | 2 | 2.484 | Peripheral plasma membrane protein CASK OS=Homo sapiens GN=CASK PE=1 SV=3 | sp\|O14936\|CSKP_HUMAN |
| 85 | 2 | 2 | 0.8571 | Hedgehog-interacting protein OS=Homo sapiens GN=HHIP PE=1 SV=3 | sp\|Q96QV1\|HHIP_HUMAN |
| 86 | 2 | 2 | 13.25 | Small integral membrane protein 10 OS=Homo sapiens GN=SMIM10 PE=4 SV=4 | sp\|Q96HG1\|SIM10_HUMAN |
| 87 | 2 | 2 | 0.9539 | Interleukin-23 receptor OS=Homo sapiens GN=IL23R PE=1 SV=3 | sp\|Q5VWK5\|IL23R_HUMAN |
| 88 | 2 | 2 | 0.8547 | Disintegrin and metalloproteinase domain-containing protein 9 OS=Homo sapiens GN=ADAM9 PE=1 SV=1 | sp\|Q13443\|ADAM9_HUMAN |
| 89 | 2 | 2 | 12.12 | Interferon-induced transmembrane protein 2 OS=Homo sapiens GN=IFITM2 PE=1 SV=2 | sp\|Q01629\|IFM2_HUMAN |
| 90 | 2 | 2 | 4.977 | Ras-related protein Rab-28 OS=Homo sapiens GN=RAB28 PE=1 SV=2 | sp\|P51157\|RAB28_HUMAN |
| 91 | 2 | 2 | 0.8159 | Neural cell adhesion molecule 1 OS=Homo sapiens GN=NCAM1 PE=1 SV=3 | sp\|P13591\|NCAM1_HUMAN |
| 92 | 2 | 2 | 2.244 | Olfactory receptor 6C75 OS=Homo sapiens GN=OR6C75 PE=3 SV=1 | sp\|A6NL08\|O6C75_HUMAN |
| 93 | 2 | 2 | 0.6744 | REVERSED Bone morphogenetic protein receptor type-2 OS=Homo sapiens GN=BMPR2 PE=1 SV=2 | RRRRRsp\|Q13873\|BMPR2_HUMAN |

**480kDa Complex**

| **N** | **Unused** | **Total** | **%Cov** | **Name** | **Accession** |
| --- | --- | --- | --- | --- | --- |
| 1 | 59.49 | 59.49 | 37.98 | Heat shock protein HSP 90-alpha OS=Homo sapiens GN=HSP90AA1 PE=1 SV=5 | sp\|P07900\|HS90A_HUMAN |
| 2 | 52.29 | 52.29 | 31.67 | Plasma membrane calcium-transporting ATPase 4 OS=Homo sapiens GN=ATP2B4 PE=1 SV=2 | sp\|P23634\|AT2B4_HUMAN |
| 3 | 47.78 | 47.78 | 44.89 | Heat shock cognate 71 kDa protein OS=Homo sapiens GN=HSPA8 PE=1 SV=1 | sp\|P11142\|HSP7C_HUMAN |
| 4 | 40.73 | 40.73 | 17.47 | Talin-1 OS=Homo sapiens GN=TLN1 PE=1 SV=3 | sp\|Q9Y490\|TLN1_HUMAN |
| 5 | 37.84 | 37.84 | 24.93 | Sodium/potassium-transporting ATPase subunit alpha-1 OS=Homo sapiens GN=ATP1A1 PE=1 SV=1 | sp\|P05023\|AT1A1_HUMAN |
| 6 | 28.52 | 28.52 | 27.11 | Transferrin receptor protein 1 OS=Homo sapiens GN=TFRC PE=1 SV=2 | sp\|P02786\|TFR1_HUMAN |
| 7 | 23.98 | 23.98 | 26.67 | 4F2 cell-surface antigen heavy chain OS=Homo sapiens GN=SLC3A2 PE=1 SV=3 | sp\|P08195\|4F2_HUMAN |
| 8 | 18.61 | 18.61 | 35.35 | Aspartate aminotransferase, mitochondrial OS=Homo sapiens GN=GOT2 PE=1 SV=3 | sp\|P00505\|AATM_HUMAN |
| 9 | 18.01 | 18.02 | 40.64 | Voltage-dependent anion-selective channel protein 1 OS=Homo sapiens GN=VDAC1 PE=1 SV=2 | sp\|P21796\|VDAC1_HUMAN |
| 10 | 17.63 | 17.81 | 17.79 | Integrin beta-1 OS=Homo sapiens GN=ITGB1 PE=1 SV=2 | sp\|P05556\|ITB1_HUMAN |
| 11 | 15.14 | 15.14 | 17.54 | Catenin beta-1 OS=Homo sapiens GN=CTNNB1 PE=1 SV=1 | sp\|P35222\|CTNB1_HUMAN |
| 12 | 15.1 | 15.13 | 17.66 | Catenin alpha-1 OS=Homo sapiens GN=CTNNA1 PE=1 SV=1 | sp\|P35221\|CTNA1_HUMAN |
| 13 | 14.71 | 14.86 | 4.924 | Nesprin-2 OS=Homo sapiens GN=SYNE2 PE=1 SV=3 | sp\|Q8WXH0\|SYNE2_HUMAN |
| 14 | 14.39 | 14.51 | 9.056 | Integrin beta-4 OS=Homo sapiens GN=ITGB4 PE=1 SV=5 | sp\|P16144\|ITB4_HUMAN |
| 15 | 14.22 | 14.37 | 31.3 | Septin-2 OS=Homo sapiens GN=SEPT2 PE=1 SV=1 | sp\|Q15019\|SEPT2_HUMAN |
| 16 | 14.16 | 14.18 | 13.12 | Alpha-actinin-1 OS=Homo sapiens GN=ACTN1 PE=1 SV=2 | sp\|P12814\|ACTN1_HUMAN |
| 17 | 13.94 | 14.05 | 11.16 | AP-2 complex subunit alpha-1 OS=Homo sapiens GN=AP2A1 PE=1 SV=3 | sp\|O95782\|AP2A1_HUMAN |
| 18 | 12 | 12 | 13.66 | ATP-binding cassette sub-family B member 6, mitochondrial OS=Homo sapiens GN=ABCB6 PE=1 SV=1 | sp\|Q9NP58\|ABCB6_HUMAN |
| 19 | 11.61 | 11.62 | 16.98 | Minor histocompatibility antigen H13 OS=Homo sapiens GN=HM13 PE=1 SV=1 | sp\|Q8TCT9\|HM13_HUMAN |
| 20 | 11.38 | 11.43 | 23.33 | ATP synthase subunit alpha, mitochondrial OS=Homo sapiens GN=ATP5A1 PE=1 SV=1 | sp\|P25705\|ATPA_HUMAN |
| 21 | 11.3 | 11.38 | 13.05 | Protein diaphanous homolog 1 OS=Homo sapiens GN=DIAPH1 PE=1 SV=2 | sp\|O60610\|DIAP1_HUMAN |
| 22 | 10 | 10 | 3.039 | Lipopolysaccharide-responsive and beige-like anchor protein OS=Homo sapiens GN=LRBA PE=1 SV=4 | sp\|P50851\|LRBA_HUMAN |
| 23 | 9.78 | 9.81 | 19.78 | Anoctamin-6 OS=Homo sapiens GN=ANO6 PE=1 SV=2 | sp\|Q4KMQ2\|ANO6_HUMAN |
| 24 | 8.99 | 9.06 | 2.289 | Prolow-density lipoprotein receptor-related protein 1 OS=Homo sapiens GN=LRP1 PE=1 SV=2 | sp\|Q07954\|LRP1_HUMAN |
| 25 | 7.96 | 7.97 | 28.11 | Alpha-enolase OS=Homo sapiens GN=ENO1 PE=1 SV=2 | sp\|P06733\|ENOA_HUMAN |
| 26 | 7.39 | 7.44 | 9.912 | Integrin alpha-6 OS=Homo sapiens GN=ITGA6 PE=1 SV=5 | sp\|P23229\|ITA6_HUMAN |
| 27 | 7.11 | 7.15 | 18.44 | Basigin OS=Homo sapiens GN=BSG PE=1 SV=2 | sp\|P35613\|BASI_HUMAN |
| 28 | 6.32 | 6.33 | 11.18 | Solute carrier family 2, facilitated glucose transporter member 1 OS=Homo sapiens GN=SLC2A1 PE=1 SV=2 | sp\|P11166\|GTR1_HUMAN |
| 29 | 6.04 | 6.04 | 51.32 | Protein deglycase DJ-1 OS=Homo sapiens GN=PARK7 PE=1 SV=2 | sp\|Q99497\|PARK7_HUMAN |
| 30 | 5.84 | 5.89 | 16.21 | Adenylyl cyclase-associated protein 1 OS=Homo sapiens GN=CAP1 PE=1 SV=5 | sp\|Q01518\|CAP1_HUMAN |
| 31 | 5.81 | 5.88 | 27.6 | Sodium/potassium-transporting ATPase subunit beta-3 OS=Homo sapiens GN=ATP1B3 PE=1 SV=1 | sp\|P54709\|AT1B3_HUMAN |
| 32 | 5.81 | 5.87 | 5.993 | E-cadherin OS=Homo sapiens PE=2 SV=1 | tr\|Q9UII7\|Q9UII7_HUMAN |
| 33 | 5.63 | 5.66 | 25.57 | Transmembrane emp24 domain-containing protein 10 OS=Homo sapiens GN=TMED10 PE=1 SV=2 | sp\|P49755\|TMEDA_HUMAN |
| 34 | 5.21 | 5.31 | 18.01 | Keratin, type II cytoskeletal 1 OS=Homo sapiens GN=KRT1 PE=1 SV=6 | sp\|P04264\|K2C1_HUMAN |
| 35 | 4.57 | 4.62 | 6.198 | Lysine--tRNA ligase OS=Homo sapiens GN=KARS PE=1 SV=3 | sp\|Q15046\|SYK_HUMAN |
| 36 | 4.55 | 4.61 | 7.364 | AP-2 complex subunit beta OS=Homo sapiens GN=AP2B1 PE=1 SV=1 | sp\|P63010\|AP2B1_HUMAN |
| 37 | 4.38 | 4.41 | 12.75 | Neutral amino acid transporter B(0) OS=Homo sapiens GN=SLC1A5 PE=1 SV=2 | sp\|Q15758\|AAAT_HUMAN |
| 38 | 4.1 | 4.11 | 7.126 | AP-2 complex subunit mu OS=Homo sapiens GN=AP2M1 PE=1 SV=2 |  |
| 39 | 4.04 | 4.05 | 19.32 | 40S ribosomal protein SA OS=Homo sapiens GN=RPSA PE=2 SV=1 | tr\|Q96RS2\|Q96RS2_HUMAN |
| 40 | 4.02 | 4.12 | 11.99 | Lysosome-associated membrane glycoprotein 1 OS=Homo sapiens GN=LAMP1 PE=1 SV=3 | sp\|P11279\|LAMP1_HUMAN |
| 41 | 4 | 4 | 6.136 | Protein disulfide-isomerase A6 OS=Homo sapiens GN=PDIA6 PE=1 SV=1 | sp\|Q15084\|PDIA6_HUMAN |
| 42 | 4 | 4 | 11.27 | AP-2 complex subunit sigma OS=Homo sapiens GN=AP2S1 PE=1 SV=2 | sp\|P53680\|AP2S1_HUMAN |
| 43 | 3.86 | 3.95 | 4.158 | Solute carrier family 12 member 9 OS=Homo sapiens GN=SLC12A9 PE=1 SV=1 | sp\|Q9BXP2\|S12A9_HUMAN |
| 44 | 3.44 | 3.51 | 7.495 | Large neutral amino acids transporter small subunit 1 OS=Homo sapiens GN=SLC7A5 PE=1 SV=2 | sp\|Q01650\|LAT1_HUMAN |
| 45 | 3.23 | 3.32 | 6.649 | Nck-associated protein 1 OS=Homo sapiens GN=NCKAP1 PE=1 SV=1 | sp\|Q9Y2A7\|NCKP1_HUMAN |
| 46 | 3.04 | 3.13 | 1.5 | Agrin OS=Homo sapiens GN=AGRN PE=1 SV=5 | sp\|O00468\|AGRIN_HUMAN |
| 47 | 2.75 | 2.81 | 8.874 | Ezrin OS=Homo sapiens GN=EZR PE=1 SV=4 | sp\|P15311\|EZRI_HUMAN |
| 48 | 2.74 | 2.8 | 9.548 | Neuroplastin OS=Homo sapiens GN=NPTN PE=1 SV=2 | sp\|Q9Y639\|NPTN_HUMAN |
| 49 | 2.35 | 2.38 | 2.264 | Extended synaptotagmin-1 OS=Homo sapiens GN=ESYT1 PE=1 SV=1 | sp\|Q9BSJ8\|ESYT1_HUMAN |
| 50 | 2.17 | 2.2 | 3.802 | Ras GTPase-activating-like protein IQGAP1 OS=Homo sapiens GN=IQGAP1 PE=1 SV=1 | sp\|P46940\|IQGA1_HUMAN |
| 51 | 2.17 | 2.19 | 9.047 | Long-chain fatty acid transport protein 6 OS=Homo sapiens GN=SLC27A6 PE=2 SV=1 | sp\|Q9Y2P4\|S27A6_HUMAN |
| 52 | 2.05 | 2.05 | 6.526 | Vinculin OS=Homo sapiens GN=VCL PE=1 SV=4 | sp\|P18206\|VINC_HUMAN |
| 53 | 2.03 | 2.03 | 0.4071 | Dystrophin OS=Homo sapiens GN=DMD PE=1 SV=3 |  |
| 54 | 2.01 | 2.01 | 7.308 | Carbonic anhydrase 2 OS=Homo sapiens GN=CA2 PE=1 SV=2 | sp\|P00918\|CAH2_HUMAN |
| 55 | 2 | 26.26 | 16.93 | Plasma membrane calcium-transporting ATPase 1 OS=Homo sapiens GN=ATP2B1 PE=1 SV=3 | sp\|P20020\|AT2B1_HUMAN |
| 56 | 2 | 2 | 1.877 | Misshapen-like kinase 1 OS=Homo sapiens GN=MINK1 PE=1 SV=2 | sp\|Q8N4C8\|MINK1_HUMAN |
| 57 | 2 | 2 | 3.523 | Leucine zipper putative tumor suppressor 1 OS=Homo sapiens GN=LZTS1 PE=1 SV=3 | sp\|Q9Y250\|LZTS1_HUMAN |
| 58 | 2 | 2 | 7.309 | Cytoskeleton-associated protein 4 OS=Homo sapiens GN=CKAP4 PE=1 SV=2 | sp\|Q07065\|CKAP4_HUMAN |
| 59 | 2 | 2 | 2.484 | Peripheral plasma membrane protein CASK OS=Homo sapiens GN=CASK PE=1 SV=3 | sp\|O14936\|CSKP_HUMAN |
| 60 | 2 | 2 | 1.099 | Fer-1-like protein 5 OS=Homo sapiens GN=FER1L5 PE=2 SV=2 | sp\|A0AVI2\|FR1L5_HUMAN |
| 61 | 2 | 2 | 0.7792 | Plexin-D1 OS=Homo sapiens GN=PLXND1 PE=1 SV=3 | sp\|Q9Y4D7\|PLXD1_HUMAN |
| 62 | 2 | 2 | 4.062 | Olfactory receptor 51E2 OS=Homo sapiens GN=OR51E2 PE=2 SV=1 | sp\|Q9H255\|O51E2_HUMAN |
| 63 | 2 | 2 | 0.8571 | Hedgehog-interacting protein OS=Homo sapiens GN=HHIP PE=1 SV=3 | sp\|Q96QV1\|HHIP_HUMAN |
| 64 | 2 | 2 | 13.25 | Small integral membrane protein 10 OS=Homo sapiens GN=SMIM10 PE=4 SV=4 | sp\|Q96HG1\|SIM10_HUMAN |
| 65 | 2 | 2 | 0.9539 | Interleukin-23 receptor OS=Homo sapiens GN=IL23R PE=1 SV=3 | sp\|Q5VWK5\|IL23R_HUMAN |
| 66 | 2 | 2 | 3.552 | HLA class I histocompatibility antigen, Cw-16 alpha chain OS=Homo sapiens GN=HLA-C PE=1 SV=1 | sp\|Q29960\|1C16_HUMAN |
| 66 | 0 | 2 | 3.591 | Putative HLA class I histocompatibility antigen, alpha chain H OS=Homo sapiens GN=HLA-H PE=5 SV=3 | sp\|P01893\|HLAH_HUMAN |
| 67 | 2 | 2 | 0.8547 | Disintegrin and metalloproteinase domain-containing protein 9 OS=Homo sapiens GN=ADAM9 PE=1 SV=1 | sp\|Q13443\|ADAM9_HUMAN |
| 68 | 2 | 2 | 1.718 | Sodium/hydrogen exchanger 1 OS=Homo sapiens GN=SLC9A1 PE=1 SV=2 | sp\|P19634\|SL9A1_HUMAN |
| 69 | 2 | 2 | 2.244 | Olfactory receptor 6C75 OS=Homo sapiens GN=OR6C75 PE=3 SV=1 | sp\|A6NL08\|O6C75_HUMAN |
| 70 | 2 | 2 | 0.6744 | REVERSED Bone morphogenetic protein receptor type-2 OS=Homo sapiens GN=BMPR2 PE=1 SV=2 | RRRRRsp\|Q13873\|BMPR2_HUMAN |

**280kDa Complex (Reactome Pathways)**

| **#term ID** | **term description** | **observed gene count** | **background gene count** | **false discovery rate** |
| --- | --- | --- | --- | --- |
| HSA-210991 | Basigin interactions | 7 | 25 | 8.91E-11 |
| HSA-382551 | Transport of small molecules | 15 | 706 | 7.32E-10 |
| HSA-109582 | Hemostasis | 12 | 601 | 2.13E-07 |
| HSA-1500931 | Cell-Cell communication | 7 | 127 | 7.47E-07 |
| HSA-446728 | Cell junction organization | 6 | 91 | 2.28E-06 |
| HSA-3000170 | Syndecan interactions | 4 | 26 | 1.82E-05 |
| HSA-1266738 | Developmental Biology | 12 | 1023 | 2.40E-05 |
| HSA-1643685 | Disease | 12 | 1018 | 2.40E-05 |
| HSA-182218 | Nef Mediated CD8 Down-regulation | 3 | 7 | 3.04E-05 |
| HSA-422475 | Axon guidance | 9 | 541 | 3.75E-05 |
| HSA-167590 | Nef Mediated CD4 Down-regulation | 3 | 9 | 4.63E-05 |
| HSA-9006934 | Signaling by Receptor Tyrosine Kinases | 8 | 437 | 6.72E-05 |
| HSA-8866427 | VLDLR internalisation and degradation | 3 | 12 | 8.17E-05 |
| HSA-373760 | L1CAM interactions | 5 | 116 | 9.37E-05 |
| HSA-5140745 | WNT5A-dependent internalization of FZD2, FZD5 and ROR2 | 3 | 13 | 9.37E-05 |
| HSA-6798695 | Neutrophil degranulation | 8 | 471 | 9.37E-05 |
| HSA-177504 | Retrograde neurotrophin signalling | 3 | 14 | 9.47E-05 |
| HSA-5099900 | WNT5A-dependent internalization of FZD4 | 3 | 15 | 0.00011 |
| HSA-8856828 | Clathrin-mediated endocytosis | 5 | 137 | 0.00015 |
| HSA-8964038 | LDL clearance | 3 | 19 | 0.00017 |
| HSA-5218920 | VEGFR2 mediated vascular permeability | 3 | 27 | 0.0004 |
| HSA-5626467 | RHO GTPases activate IQGAPs | 3 | 29 | 0.00047 |
| HSA-3000157 | Laminin interactions | 3 | 30 | 0.0005 |
| HSA-70268 | Pyruvate metabolism | 3 | 30 | 0.0005 |
| HSA-5653656 | Vesicle-mediated transport | 8 | 649 | 0.00051 |

**480kDa Complex (Reactome Pathways)**

| **#term ID** | **term description** | **observed gene count** | **background gene count** | **false discovery rate** |
| --- | --- | --- | --- | --- |
| HSA-382551 | Transport of small molecules | 14 | 706 | 2.25E-08 |
| HSA-446728 | Cell junction organization | 7 | 91 | 1.68E-07 |
| HSA-210991 | Basigin interactions | 5 | 25 | 4.09E-07 |
| HSA-3000170 | Syndecan interactions | 4 | 26 | 2.57E-05 |
| HSA-1266738 | Developmental Biology | 12 | 1023 | 3.39E-05 |
| HSA-182218 | Nef Mediated CD8 Down-regulation | 3 | 7 | 4.38E-05 |
| HSA-422475 | Axon guidance | 9 | 541 | 5.20E-05 |
| HSA-167590 | Nef Mediated CD4 Down-regulation | 3 | 9 | 6.23E-05 |
| HSA-9006934 | Signaling by Receptor Tyrosine Kinases | 8 | 437 | 8.82E-05 |
| HSA-109582 | Hemostasis | 9 | 601 | 8.86E-05 |
| HSA-8866427 | VLDLR internalisation and degradation | 3 | 12 | 9.62E-05 |
| HSA-177504 | Retrograde neurotrophin signalling | 3 | 14 | 0.00011 |
| HSA-373760 | L1CAM interactions | 5 | 116 | 0.00011 |
| HSA-5140745 | WNT5A-dependent internalization of FZD2, FZD5 and ROR2 | 3 | 13 | 0.00011 |
| HSA-5099900 | WNT5A-dependent internalization of FZD4 | 3 | 15 | 0.00013 |
| HSA-5663205 | Infectious disease | 7 | 363 | 0.00016 |
| HSA-8856828 | Clathrin-mediated endocytosis | 5 | 137 | 0.00016 |
| HSA-8964038 | LDL clearance | 3 | 19 | 0.00018 |
| HSA-1474244 | Extracellular matrix organization | 6 | 298 | 0.0004 |
| HSA-1643685 | Disease | 10 | 1018 | 0.0004 |
| HSA-216083 | Integrin cell surface interactions | 4 | 83 | 0.0004 |
| HSA-5218920 | VEGFR2 mediated vascular permeability | 3 | 27 | 0.0004 |
| HSA-5626467 | RHO GTPases activate IQGAPs | 3 | 29 | 0.00044 |
| HSA-3000157 | Laminin interactions | 3 | 30 | 0.00047 |
| HSA-418990 | Adherens junctions interactions | 3 | 32 | 0.0005 |

**Supplemental Table ST3.** Demographic data.

|  |  | **Maternal serum sample** (11-14 weeks of gestation) | | | | | |  |
| --- | --- | --- | --- | --- | --- | --- | --- | --- |
|  |  | ***Control group (N=52)*** | | ***PE group (N=26)*** | | | | ***P value*** |
|  |  |  | |  | | | |  |
| **Age of women (years)** |  | 35.29(24-44) | | 34.96 (27-45) | | | | 0.749 |
| **Gravidity** |  | 1.81 (1-7) | | 1.88 (1-7) | | | | 0.806 |
| **Parity** |  | 0.44 (0-2) | | 0.58 (0-4) | | | | 0.505 |
| **BMI** |  | 22.61 (16.65- 34.48) | | 25.06 (18.22-37.48) | | | | 0.247 |
| **Gestational age at birth (weeks)** |  | 37.23 (30-41) | | 36.77 (29-41) | | | | 0.417 |
| **Fetal birth weight (g)** |  | 2760(1195-3917) | | 2410(930-3970) | | | | 0.015 |
| **Placental weight (g)** |  | 583(285-1065) | | 476(253-1108) | | | | 0.025 |
| **Blood pressure, MAP at booking (mmHg)** |  | 83.17 (68.3-100) | | 92.5 (77-114) | | | | 0.003 |
| **Spot urine protein (g/L)** |  | 0.04 (0-0.04) | | 0.54 (0.06-13.85) | | | | 0.001 |
| **CD147 serum level**  **(Mean ± SD, pg/mL)** |  | 88.94 ± 18.94  (58.2-143.5) | | 80.04 ± 14.52  (54.3-114.8) | | | | 0.025 |
|  |  |  | | *Early onset*  (n=8) | *Late onset*  (n=18) | | |  |
|  |  | 88.94 ± 18.94  (58.2-143.5) | | 74.15 ± 15.48  (54.3-98.8) | 82.66 ± 13.7  (57.0-114.8) | | | 0.034 (early onset vs control)  0.140 (late onset vs control)  0.205 (early onset vs late onset) |
|  |  | |  | | |  |  | |
